# Nature-derived materials for the fabrication of functional biodevices

**DOI:** 10.1016/j.mtbio.2020.100065

**Published:** 2020-06-12

**Authors:** S. Pradhan, A.K. Brooks, V.K. Yadavalli

**Affiliations:** Department of Chemical and Life Science Engineering, Virginia Commonwealth University, Richmond, VA, 23284, USA

**Keywords:** Biomaterials, Silk, Chitin, Cellulose, Flexible bioelectronics, Nature-inspired

## Abstract

Nature provides an incredible source of inspiration, structural concepts, and materials toward applications to improve the lives of people around the world, while preserving ecosystems, and addressing environmental sustainability. In particular, materials derived from animal and plant sources can provide low-cost, renewable building blocks for such applications. Nature-derived materials are of interest for their properties of biodegradability, bioconformability, biorecognition, self-repair, and stimuli response. While long used in tissue engineering and regenerative medicine, their use in functional devices such as (bio)electronics, sensors, and optical systems for healthcare and biomonitoring is finding increasing attention. The objective of this review is to cover the varied nature derived and sourced materials currently used in active biodevices and components that possess electrical or electronic behavior. We discuss materials ranging from proteins and polypeptides such as silk and collagen, polysaccharides including chitin and cellulose, to seaweed derived biomaterials, and DNA. These materials may be used as passive substrates or support architectures and often, as the functional elements either by themselves or as biocomposites. We further discuss natural pigments such as melanin and indigo that serve as active elements in devices. Increasingly, combinations of different biomaterials are being used to address the challenges of fabrication and performance in human monitoring or medicine. Finally, this review gives perspectives on the sourcing, processing, degradation, and biocompatibility of these materials. This rapidly growing multidisciplinary area of research will be advanced by a systematic understanding of nature-inspired materials and design concepts in (bio)electronic devices.

## Graphical abstract

**Figure:**
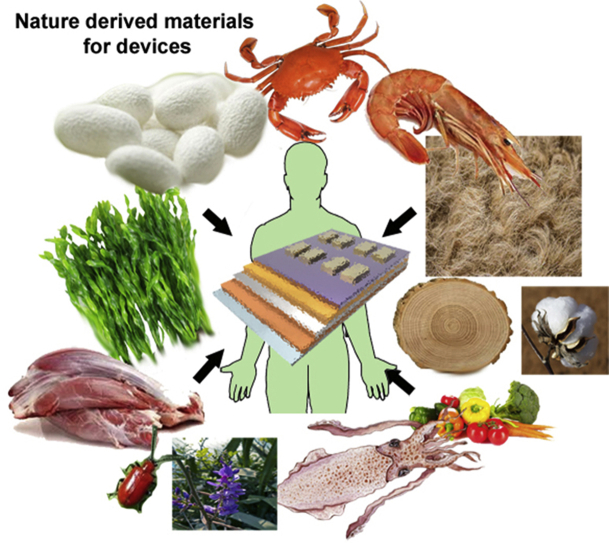

## 1 Introduction

**Fig. 1:**
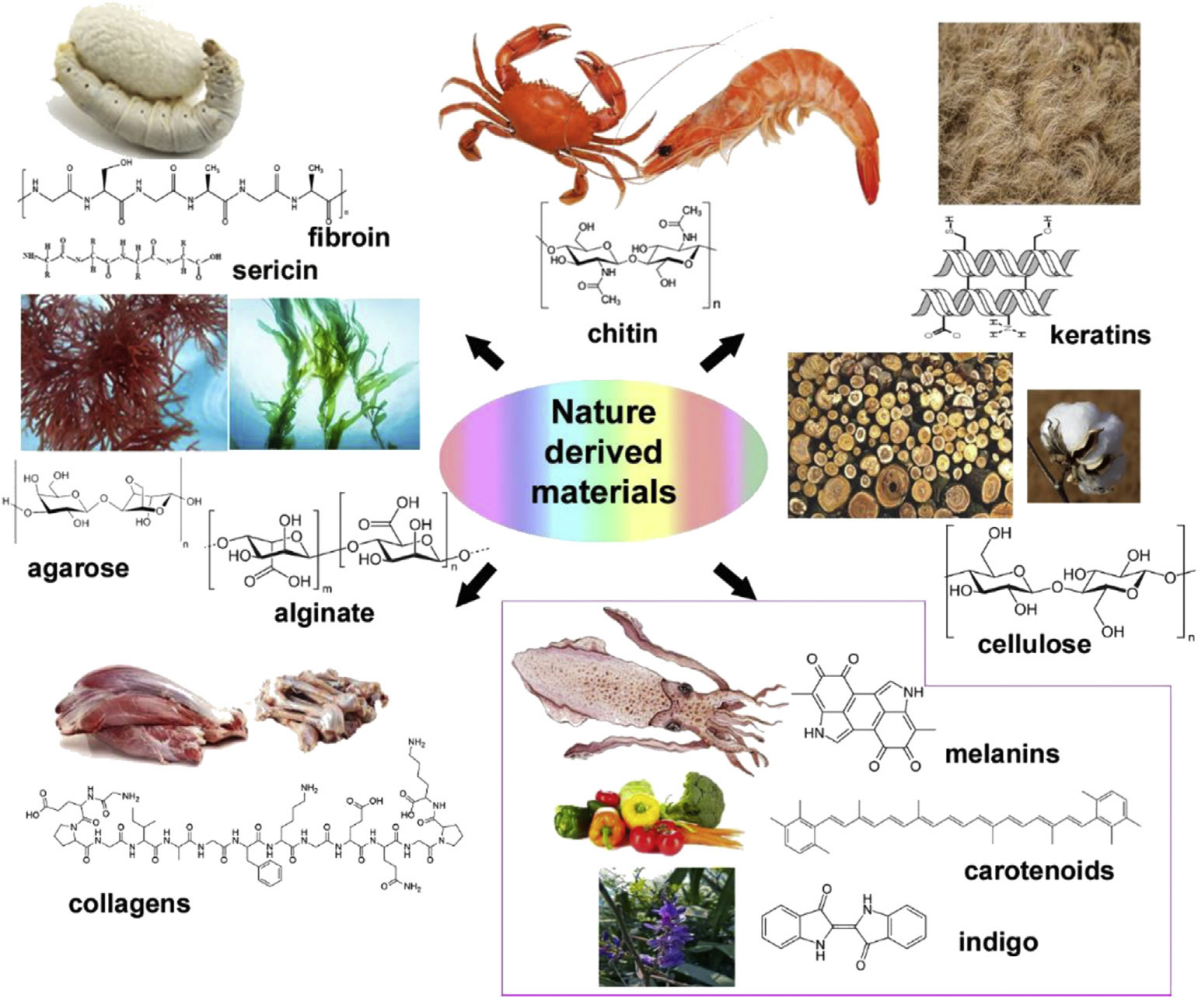
Sources of some of the natural materials discussed in this work. The materials can serve as both the structural supports or the active components of various devices and in some cases the entire device can be fabricated using combinations of nature-derived materials. The box in the lower right shows pigments that can serve as organic semiconducting materials.

Hierarchical architectures in natural materials display fundamental structural and bioactive properties that have evolved over millions of years. They may possess combinations of several functions including bioactivity, mechanical toughness, optical or electromagnetic properties, and biocompatibility, either by themselves, or as composites [1,2]. Owing to this palette of properties, there has been extensive research toward using natural biomaterials for a variety of tissue engineering and regenerative medicine applications [3,4]. These include natural and bioinspired materials, derived from animal and plant sources, either directly, or with minimal extraction and processing (Fig. 1). Being renewable and degradable, they can provide low-cost, sustainable building blocks while conferring benefits such as bioconformability, biorecognition, self-repair, and stimuli response [5,6]. Their biological activity, porosity, and mechanical properties can be tuned by changing polymerization conditions or chemical functionalization [7]. Some materials can directly contact tissue or skin, or be implanted without adverse effects. Others can biodegrade *in vivo* implying that they can dissolve or resorb safely into the body at controlled rates, enabling their use in different biomedical applications [8].

Recently, there has been a great interest in adapting nature-derived materials for the formation of active functional devices such as (bio)electronics, sensors, and optical systems. In many of these cases, natural biomaterials serve as the *structural* or the *functional* component, and in some cases, both. They may be used either as a pure material or as a composite. Such devices have been formed in various configurations, including rigid or mechanically flexible forms. Materials that are flexible and conformable with skin or soft tissue can be used as ‘electronic skins,’ soft-robotics, and human–machine interfaces [[9], [10], [11], [12], [13]]. The properties that make them attractive for regenerative medicine, such as tunable mechanical strength and flexibility, lend the materials for adoption in flexible and wearable devices. For several applications, particularly those that interact and interface with biological systems, there is a strong need for materials with such unique characteristics [14,15]. While possessing structural and material compliance, it is desirable that they be mechanically robust while providing required function [[16], [17], [18]]. They may be combined with other functional materials to impart electrochemical properties. Nanofillers can be incorporated into (bio)polymers to form composites to augment their performance [19,20].

There has been another impetus for the interest around natural materials for device fabrication, driven by an increasing desire for reducing ‘e-waste.’ Waste from electronic equipment is estimated to be in excess of 50 million tons per year. This necessitates adopting sustainable materials and processes, while reinterpreting the product life cycle [6]. Discarded e-waste often comprises non-degradable polymers and/or heavy metals, which can be damaging to the environment. Several conventional devices are based on scarce natural elements (e.g. Ga, Ge, In), which are susceptible to exhaustion, or other economic considerations. Biodegradable natural materials may provide a solution as renewable resources that can be degraded, dissolved, or composted into the surrounding environment [21]. Biodegradation can also cover devices that are specifically designed to degrade *in vivo* (viz. transient devices) [22]. Another driving force lies in the use of ‘green processing.’ This endows the electronics with low carbon-footprint technology, environmental safety and disposability, potentially decreasing the energy costs for fabrication and recycling operations, as well as health risks associated with harmful processing.

This review is intended to cover the recent advances in the area of using nature-derived materials for the fabrication of functional devices. We use the terms ‘natural’ and ‘nature-derived’ interchangeably (the term ‘natural-origin’ is sometimes used in this context). The goal is to draw a distinction to synthetic or man-made materials. Systems in which biodegradable and biocompatible synthetic polymers are being used in soft electronics and transient devices have been previously reviewed [7,23]. The use of organic and inorganic materials specifically for soft electronics has been covered in a comprehensive review [24]. The use of functional biomaterials toward flexible electronics and sensors was covered by Sun et al. [25]. Using nature-inspired structural materials for flexible electronics has been covered elegantly by Liu et al. [26]. In these earlier reviews, the focus was on structural design strategies based on hybridizing several classes of materials (organic and inorganic, synthetic, and natural), both rigid or soft, on the same device platform. Here, our objective is to cover active biodevices fabricated wholly or in part using nature-derived materials (serving as the structural and/or the functional component). By ‘active’ devices, we imply those that primarily possess electrical or electronic behavior, such as bioelectronics, optoelectronics, sensors, and energy storage devices. Our search parameters to comprehensively cover the literature included materials of interest (e.g. ‘silk,’ ‘collagen,’ ‘agarose,’ ‘nature-derived,’ etc.), together with terms such as ‘sensor,’ ‘bioelectronics,’ ‘device,’ ‘functional,’ and ‘conducting’ and attributes such as ‘biodegradable’ and ‘flexible.’

Devices may be in both flexible or non-flexible (rigid) formats. In many cases, the natural biomaterial may primarily serve as a passive substrate or support for the active architectures. Thus, applications such as bioinspired adhesives [27], photonics [28], or surface modification (e.g. superhydrophobicity) [29] are beyond the purview of this review. We peripherally consider cases of active tissue scaffolds in which for instance, natural materials are used in conjunction with electroactive materials to promote cell growth, differentiation, or for recording signals [30]. For applications involving tissue engineering or drug delivery using nature-derived biomaterials, the reader is referred to other reviews (e.g. Refs. [4,[31], [32], [33]]).

**Fig. 2:**
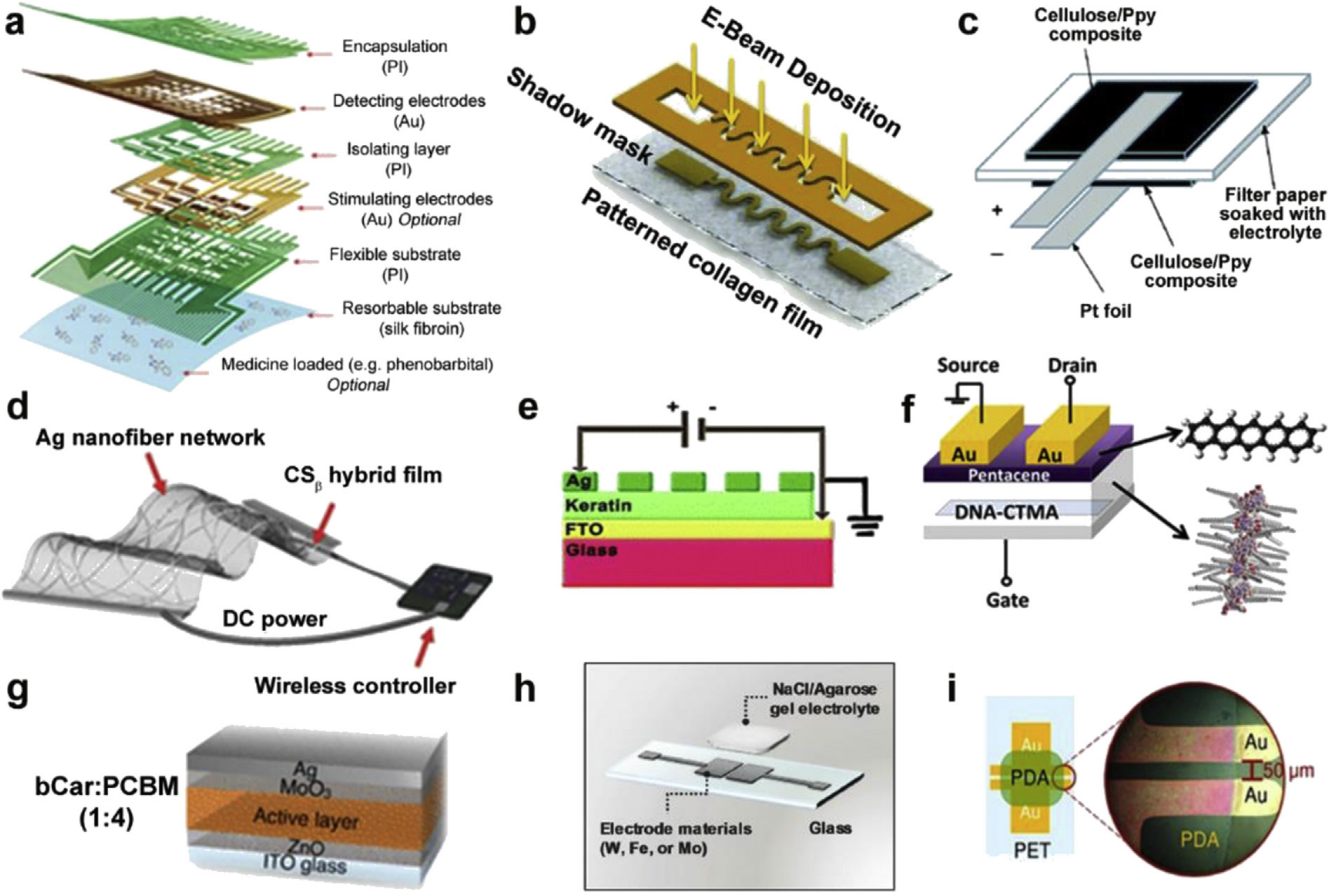
**Schematics of various forms of use**. **(a)** Device configuration of silk-enabled conformal multifunctional bioelectronics for the investigation of spatiotemporal epileptiform activities and multimodal neural encoding/decoding [66]. **(b)** Metallic patterns were deposited on collagen film using E-beam evaporation through a shadow mask [94]. **(c)** A cellulose--PPy conductive paper composite as an ultrafast battery [140]. **(d)** Skin-attachable wireless film heater with an AgNF network on the CS_β_31 hybrid film (scale bar = 100 μm) [162]. **(e)** A fabricated Ag/human hair keratin/FTO configuration for physically transient resistive switching memory devices [77]. **(f)** Schematic configuration of an OFET memory device and chemical structure of DNA–CTMA and pentacene [235]. **(g)** Ultrathin organic solar cells with β-carotene as an electron donor [272]. (**h**) A planar-type supercapacitor consisting of biodegradable metal thin-film (W, Fe, or Mo) electrodes and an NaCl/agarose gel electrolyte on a glass substrate [196]. (**i**) Moisture sensor based on self-assembled dopamine–melanin thin films [257]. (*All images used with permission*).

This review is organized by material focused on nature-derived biomaterials, including polypeptides (e.g. silk, collagen) and polysaccharides (e.g. chitin, chitosan). We discuss examples in which the biomaterial may be used as a passive substrate for other active elements, or as a functional layer or structure in and of itself. (Some example device schematics are shown in Fig. 2, showing the wide diversity of their use.) Given that many of the nature-derived materials are dielectrics, in this latter embodiment, they may be combined with metals, nanotubes, organic semiconductors, or conducting polymers to form active composites. We discuss aspects of such composites that make them advantageous. Finally, we cover some examples of nature-derived conductors and semiconductors such as the pigments melanin and indigo that can provide alternatives to synthetics. Through this review, we intend to show the diversity of functional devices that can be formed using nature-derived biomaterials, and demonstrate their viability and competitiveness for real-world applications as opposed to novelty research.

## 2 Proteins

### 2.1 Silk proteins

**Fig. 3:**
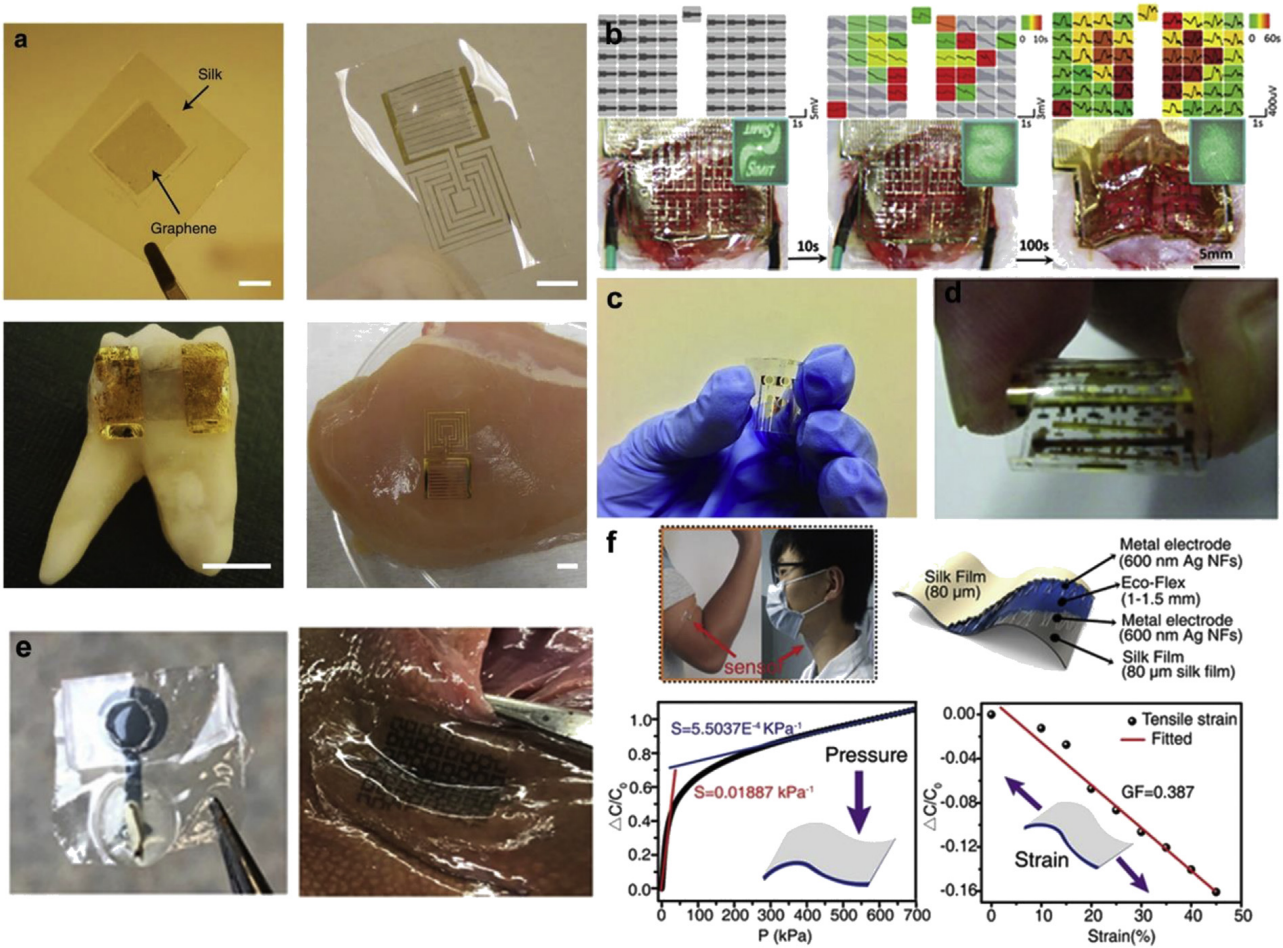
**Silk-based devices**. **(a)** Graphene printed on bioresorbable silk film to form a passive wireless telemetry system consisting of a planar meander line inductor and interdigitated capacitive electrodes. Sensor transferred onto the surface of a human molar and muscle tissue. Scale bars are 5 mm [45]. **(b)** Silk-enabled conformal multifunctional bioelectronics for spatiotemporal epileptiform activities and multimodal neural encoding/decoding. Device images and ECoG signals during conformal mounting progress on rat brain; left, middle, and right columns, respectively, show status before, during (promoted by adding normal saline), and after compliant mounting. Colors of signal lattices are associated with the time experienced to provide effective electrocorticography (ECoG) signals (gray, non-contact) [66]. **(c)** Sericin printed on flexible PET substrate to form a flexible memory device [67]. **(d)** Rollable pentacene OTFT with silk fibroin as the gate dielectric [291]. **(e)** Sericin--PEDOT:PSS ink printed on a flexible fibroin substrate and used for the detection of VEGF [69]. **(f)** Stretchable sensor directly laminated onto human skins for monitoring the movements of elbow and laryngeal. The sandwich structure of the pressure–strain sensor. The relationship between the variation of capacitance and the applied pressure and between the variation of capacitance and the applied tensile strain [63]. (*All images used with permission*).

Silk is mainly derived from domesticated *Bombyx mori* silkworm cocoons, which have been farmed for almost 5,000 years. Various non-mulberry species of silkworms have also been reported for their interesting properties [34]. Proteins obtained from silkworms provide an ensemble of properties to address the challenges of fabricating functional devices. Silk exists as a self-assembled fiber, consisting of a core protein fibroin (70%), surrounded by a glue glycoprotein sericin (30%) [35]. These proteins can degrade controllably over time, via proteolytic degradation and resorption without an adverse immunogenic response [36,37]. Typically, when fibroin and sericin are completely separated, each induce only mild or minimal immunological reactions on implantation [38]. Although extensive reports have focused on the use of silk proteins in drug delivery, nanostructured scaffolds, and regenerative medicine, recent works have shown the way toward applications in photonics, bioelectronics, and devices without the rigorous processing involved with synthetic materials [[39], [40], [41]]. In this section, we discuss some functional devices using silk proteins as the structural and/or functional element. Some representative devices featuring silk proteins are shown in Fig. 3.

Silk fibroin (SF) has primarily been used as a support substrate in a variety of devices. Excellent reviews on the use of silk in (bio)electronics have been reported [42,43]. Functional components, such as nanowires, conductive polymers (CPs), and carbon-based materials, have been printed on fibroin substrates to create devices with improved electrical conductivity and sensitivity, while retaining mechanical properties compatible with native tissue. To improve surface conformation, an Au/Cr electrode array fabricated on a carrier wafer consisting of polyimide, poly (methyl methacrylate), and silicon (PI/PMMA/Si) was transferred to a silk film, then bonded to an anisotropic conductive film for connection to external systems. The device was placed on an exposed brain, and flushed with saline to dissolve the silk film. Decreasing the thickness of silk layer, and using a mesh electrode architecture improved conformal contact and sensing [44]. Other examples of wires printed on conformable silk substrates have been used to make sensors for food and bacteria [45,46]. Silver nanowires (AgNWs) have been patterned on SF to create capacitance-based humidity sensors to monitor human breathing [47].

Silk's degradability and mechanical softness have been used for a remotely controlled, implantable device to counter *Staphylococcus aureus* infection. A silk overcoat and substrate housed an Mg resistor and power-receiving coil, and the implant was heated wirelessly via near-field coupling. The device partially degraded in 7 days, and fully by 14 days. To improve therapeutic ability, an ampicillin/silk layer was added. Increased temperature and lower silk molecular weight led to faster release [48]. Crystalline silicon nanomembrane transistors were fabricated on a carrier wafer, then lifted onto a poly (dimethylsiloxane) (PDMS) stamp and transfer printed to flexible silk films. Complete dissolution occurred in water at room temperature within 3 min, and the still-functional transistors could be recovered using filter paper. Devices consisting of doped Si, SiO_2_, and Au encapsulated with PI were implanted subcutaneously in mice. In 2 weeks, 15–20% of the films dissolved without inflammation around the implant site [49]. Printed silk-fibroin–based triboelectric nanogenerators have used silk for its properties of transparency and flexibility. PDMS covered with a screen-printed graphite microscale pattern electrode and an SF film on top could harvest human biomechanical energy, and monitor human breathing states and motion based on passive capacitance [50].

While silk thin films are uniform layers of solution that have been cast and dried, silk fabrics consist of woven or matted silk strands. In contrast to thin fibroin films (above), silk fabric has been used as a free-standing substrate for CPs such as poly (3,4-ethylenedioxythiophene):polystyrene sulfonate (PEDOT:PSS), and multiwalled carbon nanotubes (MWCNTs) with polypyrrole (PPy) electropolymerized *in-situ*. These materials were coated in gel electrolyte and sandwiched in a cellulose film to make a supercapacitor [51]. Coated silk fabrics offer a method to enhance electrical and mechanical properties. Multifunctional silk textiles were spray-coated with biomimetic leaf-like MXene (transition metal carbide/carbonitride)/AgNW nanostructures for electromagnetic interference shielding and humidity monitoring [52]. Silk fabrics with nickel-deposited electrodes have been spray-coated with graphene oxide (GO) to create sensors for respiration monitoring. The fabrics could tolerate repetitions of bending and twisting, and sense both fast and slow breathing [53]. GO-coated silk fabric has also been used in strain sensors. The textiles could conform to the human body, where sensitivity and resistance vary with angles during stretching. The change in resistance, Δ*R*/*R*_0_, detected differences in motions such as eye blinking, mouth moving, crying, and finger bending [54]. Copper oxide nanoparticles embedded in carbon spheres were grown on carbonized silk fabrics, creating an electroconductive material for the non-enzymatic sensing of glucose [55]. Silk fabric was grafted with beta-cyclodextrin, and coated in PPy to promote self-healing behavior and retention of conductivity [56].

SF has been used as a ‘sandwich’ component for transient electronics with biodegradability and flexible resistive memory. In capacitors, it serves as a dielectric layer between two conductive layers. A tungsten/SF/magnesium sandwich, with W as the inert electrode, Mg as the active electrode, and SF as a switching layer, on polyethylene terephthalate (PET) or Si/SiO_2_ exhibited stable bipolar resistive switching behavior. The device fully disappeared in phosphate-buffered saline (PBS) at 37˚C after 24 h [57]. A photo-responsive nanocomposite film with three layers, a photo-bending layer (azobenzene incorporated PDMS), SF support, and a conductive layer of AgNWs, could function as an electrical switch. This flexible, transparent film changed with light because of the azobenzene changing molecular arrangement on irradiation, which altered the contact of the AgNWs and thus electrical conductivity between electrodes [58].

Recently, there has been a great interest in combining silk proteins with other materials to form functional composites. Composites with CPs have only recently begun to be explored. An early example showed that the PEDOT:PSS electrochemically combined with a silk thread from the natural *B. mori*. The addition of glycerol improved the conductivity, while also improving its durability in water and washing cycles [59]. Electroconductive silk fabrics have been created using SF/PPy fibers. A dissolution technique via calcium salt treatment was used to create microgaps in SF fibers, increasing the PPy content. This gave the normally compact and smooth fibers a rough and hairy structure, increasing hydrophobicity and electroconductivity. These materials showed touch sensitivity, and could be used to power a light or for electric heating [60]. For touchscreens and displays, many biomaterials are low-cost and flexible, but have low stability and mechanical integrity. A suspension of SF and laponite, a layered silicate material, mixed with KCl at basic pH was used to make recyclable films with high electrical sensitivity even after 2,000 bending cycles. These films were 85% transparent, thermally stable up to 278˚C, and their crystalline nature prevented degradation in an aqueous environment. This material was made into a touchscreen than retained functionality even at a bending angle of 180° [61].

Silk composites have been used to make devices for the detection and release of biomarkers. A nitrogen-doped SF carbon matrix was used for the detection of electroactive rutin (vitamin P) using a flexible 3-electrode system [62]. As a flexible and stretchable pressure-strain sensor, SF was doped with glycerol and steam annealed, then silver nanofibers (AgNFs) were added by electrostatic spinning and sputtering. Ecoflex™ was used as the dielectric layer between two Ag NFs/SF electrodes. This device could monitor small movements such as breathing, as well as large movements, as the capacitance changed with strain and pressure. It was stable under strain, but decomposed in 5% papain solution at 37˚C for 24 h, mimicking degradation in physiological conditions [63]. AgNW/fibroin composite fibers have been fabricated using a wet-spinning method. The composite fibers exhibited high flexibility, whereas many fibroin films are brittle, and could be folded inward and outward without losing conductivity [64]. Nitrogen-doped silk carbon textiles have been used to detect six biomarkers (glucose, lactate, AA, UA, Na^+^, and K^+^). These devices were integrated with signal collection and transmission, and nickel conductive tape or the respective electrochemical sensors were drop-casted on the fabric [65]. Silk films have been used for controlled smart-release for epilepsy therapeutics. A multilayered device was created with SF loaded with phenobarbital and alternating layers of PI and Au as various isolating and electrode layers. The device was loaded onto a rat brain. When seizure was induced, the device recorded electrical signals, which triggered drug release and caused a holographic pattern to dissolve [66].

Although most of the work with silk in devices has been reported using fibroin, progress has also been achieved using silk sericin, the water-soluble protein commonly discarded during processing. Resistance switching characteristics of a sericin protein film was demonstrated for non-volatile memory. Prototype memory devices based on sericin were built on flexible PET substrates. Significant memory performance with large resistance OFF/ON ratio (>10^6^) and long retention time (>10^3^ s) was obtained in the Ag/sericin protein/Au devices [67]. PEDOT:PSS was combined with water soluble photoreactive silk sericin to form an ink that can be micropatterned via photolithography. This ink was covalently attached to an SF film to form a flexible, free-standing sensor that could be proteolytically degraded over the course of weeks. Mechanical, electrical, and electrochemical properties could be tuned by varying the concentrations of each component. Sensing for dopamine, ascorbic acid, and glucose was shown *in vitro* [68]. This system was also used for sensing of the biomarker vascular endothelial growth factor (VEGF), whose elevated levels in blood serum can be an indicator for cancers. Anti-VEGF antibodies were embedded in the PEDOT:PSS-sericin ink that formed the electrode, and the resulting film device could stand on its own, as well as adhere to soft tissue. The device was sensitive (pg/mL) and consistent in biological fluids including buffer, serum, and urine [69].

An approach to use organic materials while addressing the low mechanical strength of PEDOT:PSS is as core-sheath conductive wires, which can be used as flexible connectors. SF functions as an insulating sheath, and was connected to a PEDOT:PSS-silk sericin electrode biosensor. The sheath insulates the fibers in the nonconductive direction without changing conductivity along the wire. This free-standing device could be linked to an external interface without metal connections, and was proteolytically biodegraded in weeks. These fibers have also been integrated with a 3-electrode organic (O3E) biosensor, maintaining a linear response to ascorbic acid (AA) over the course of 1 week and losing macroscale structural integrity over 1 month. Sensor fouling from non-specific absorption of proteins was tested, with the O3E biosensor maintaining a linear response to both AA and glucose in 40 mg/mL bovine serum albumin solution and the control solution of PBS [70,71].

### 2.2 Keratin

Keratin is a structural protein that is naturally present in a variety of organisms, particularly in epidermal cells. It is the main component of structures such as hair, feathers, hooves, horns, nails, and claws. Major sources of keratin waste include slaughterhouses (e.g. chicken feathers, beaks), the wool and leather industries (e.g. hair, hooves), and barbershops (e.g. human hair) [72]. As eventually biodegradable, keratin waste tends to degrade slowly. Consequently, these animal by-products are usually considered hazardous waste and, in many cases, environmental pollutants [73]. Various disposal options are either themselves polluting (e.g. incineration) or chemically intensive (e.g. alkali hydrolysis). However, the abundance and low cost, combined with physical strength and chemical properties, make it an interesting and viable candidate for green device applications.

Keratin's molecular and hierarchical features contribute to its characteristic strength and durability. Coiled protein strands twist together to form rope-like structures. This contributes to function as an intermediate filament in cells, where it provides structural support. α-Keratin is found in vertebrates, and has a tightly coiled helical structure that adds to the strength of hair, wool, and hooves. β-Keratin in reptiles and birds, consists of sheets that stack and form structures such as claws and beaks. Its amino acid content varies, but a relatively large number of cysteine residues provide interchain disulfide bridges. Keratin self-assembles into bundled fibers, then covalent links from disulfide bridges and H-bonding between the chains further increases strength and leads to insolubility in water [74]. It is also not easily degraded by common proteolytic enzymes such as pepsin, but can be controllably degraded via keratinases, microbes, or certain alkaline solutions. While this hinders easy processability, keratin's insolubility can be advantageous in the fabrication of multilayered devices, as it will not dissolve with the addition of solutions used to form subsequent layers. Insolubility is also desirable for applications where long-term stability is necessary [75]. Alternatively, the reduction process to remove keratin from natural sources such as hair with thioglycolic acid or sodium sulfite can break disulfide bonds, resulting in water solubility [76,77]. This shows promise for transient device applications.

**Fig. 4:**
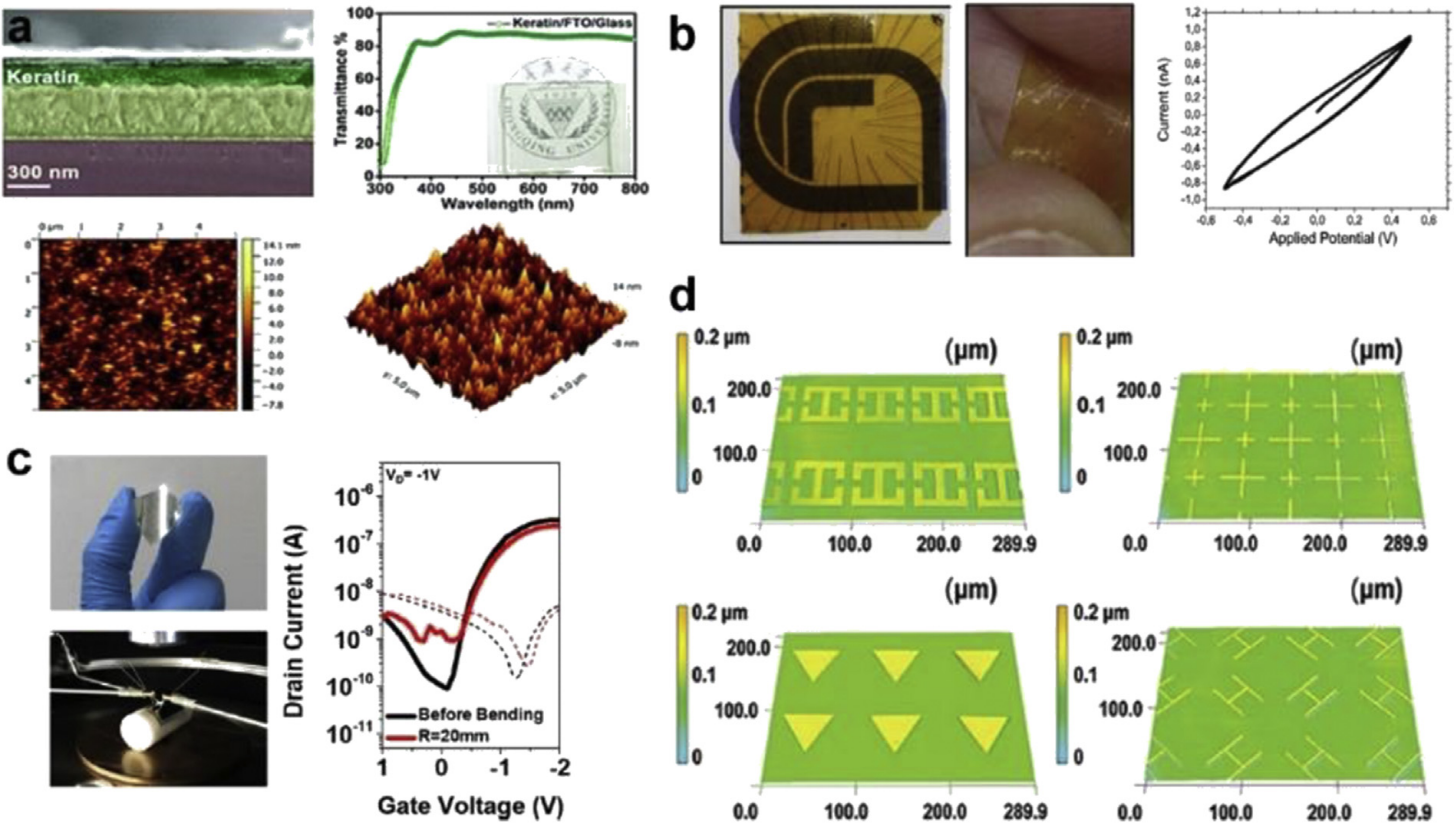
**Keratin-based devices**. **(a)** A fabricated Ag/keratin/FTO configuration showing cross-sectional scanning electron microsope (SEM), transmission spectrum and the photograph (inset) of the keratin thin film. The atomic force microscope (AFM) topography and 3D image of the keratin thin film [77]. **(b)** Keratin as a bendable all-biopolymeric microelectrode array humidity sensor. Free-standing insoluble keratin-based microelectrode array showing transparency. Bending of the sensor and cyclic voltammetry at constant 60% relative humidity [79]. **(c)** Optical image and bending test of PCDTPT/keratin flexible thin-film transistors (TFTs) on PAR films. Transfer characteristics under an applied drain voltage of −1 V, and a gate voltage of +1 to −2 V (size of device: 2 × 2 cm). **(d)** Water-based photolithography process to form high-resolution wool keratin micropatterns showing 3D confocal laser scanning microscopy images [82] (*All images used with permission*).

Keratin has mostly been used as a substrate for device fabrication. Some representative devices featuring keratin are shown in Fig. 4. Wool keratin has been reported as a substrate in a humidity sensor. It was drop-cast on top of two electrode patterns (interdigitated and spiral), forming a surface that was fairly porous and rough. The capacitance increased with relative humidity, and decreased at an even faster rate with increasing frequency. The rectangular spiral sensor had higher sensitivity and capacitance than the interdigitated sensor, owing to more edges creating ineffective zones at corners [78]. Keratin has been used as both substrate and active material for electrical transduction for humidity monitoring. Soluble diode-like keratin-based bipole (KB) sensors were created on indium tin oxide (ITO)-coated glass as an active sensor layer, with a top gold or Pd electrode as the counter-electrode. The keratin was doped with glycerol to improve the mechanical stability. Insoluble diode-like KB sensors were created by a similar process, but the addition of glutaraldehyde rendered them insoluble. The insoluble sensors had inhibited ion transfer, and much higher resistance with increasing relative humidity. 9 wt% melanin-doped keratin was made for a higher ion-conductivity composite. As ammonia had to be added to stabilize the melanin, protein folding was affected and ion-conductivity decreased. A bendable multi-electrode array was made with soluble keratin on a gold electrode, rendered insoluble by glutaraldehyde treatment, and cyclic voltammetry measured at 60% relative humidity [79].

Human hair keratin has been used in resistive switching memory devices. An Ag/keratin/ITO sandwich was made on an Si/SiO_2_ substrate. Fluorine-doped tin oxide (FTO) and Ti were tested as the bottom electrode. The device was stable for over 150 cycles with a high retention time. Ag and graphite nanoparticles were then doped in keratin intermediate layer, which increased the conductivity of the dielectric layer, and reduced the switching ratio and memory performance [76]. Transient resistive switching random access memory (RAM) device films have been created using glass, FTO as the bottom electrode, keratin as the electrolyte layer, and Ag as the top electrode. The resistance state could be switched on applying an electric field. The switching mechanism was due to the formation and rupture of Ag conductive bridges mediated by redox reactions. Thioglycolic acid was used to deoxygenate the human hair that the keratin in this device was derived from, which also destroyed its cysteine bonds. As a result, this device dissolved in water in 30 min, showing the potential transience of keratin [77].

In other degradable systems, human hair keratin was used as both a substrate and a dielectric layer for flexible organic thin-film transistors (OTFTs) based on the donor–acceptor type (D–A) conjugated polymer poly [N-9′-heptadecanyl-2,7-carbazole-alt-5,5-(4′,7′-di-2-thienyl-2′,1′,3′-benzothiadiazole)] (PCDTPT). The devices retained electrical stability under bending strain. An artificial electronic synaptic PCDTPT/keratin transistor was fabricated that exhibited memory effects via proton conduction in keratin. All these devices were degradable in an ammonium hydroxide solution [80]. Keratin extracted from chicken feathers was used as a gate dielectric in an OTFT. Many synthetic polymers used as gate dielectrics are soluble in common organic solvents, so the deposition of organic semiconductor materials will wash them away. Therefore, keratin's insolubility is advantageous in this regard. Metal-insulator-metal devices were fabricated using Si as a substrate with layers of Al, keratin, and Al pads. An OTFT was also fabricated with poly (3-hexylthiophene) (P3HT), a semiconducting polymer, as the semiconductor layer and keratin as dielectric. To demonstrate the biodegradability of keratin and potential for reducing e-waste, Si wafers, one coated in keratin and one without keratin, were submerged in an aquarium. Fish readily ate the keratin protein, and ignored the plain Si wafer [75].

Finally, keratin can be blended with other materials to form composites. To mimic the keratin--elastin composition of human skin, human hair keratin was combined with thermoplastic polyurethane to form a composite with surface roughness, flexibility, and rheological behavior similar to human skin. A single triboelectric nanogenerator was created using the composite film, which gave output voltages in the range of 12–80 V/cm^2^ when contacted with materials ranging from a human finger to a PTFE sheet to aluminum foil [81]. Recently, new methods of micropatterning keratin have been developed using photolithography, which can open up applications in device fabrication. Wool keratin was functionalized with photoreactive 2-isocyanatoethyl methacrylate to form photoresist-like wool keratin. Using photolithography, high-resolution micropatterns could be formed. The patterned films were stable in PBS over 7 days, and degradable in 15 days with the addition of protease. Cell studies showed preferential adhesion to the films due to wool keratin's natural RGD (Arg–Gly–Asp) adhesive ligands with no noticeable cytotoxicity [82].

### 2.3 Collagen and gelatin

Collagen is the most abundant protein on earth, found in the extracellular matrix of connective tissues [83]. Its main function is to provide structural and biomechanical support in bone, cartilage, skin, tendon, and ligaments. Collagen has a triple-helical structure comprising three polypeptide chains [84]. It is a hierarchical biomaterial with varying structural and morphological properties across length scales [85]. There are at least 28 genetically distinct types of collagen based on their complexity and diversity in structure and function [86]. Collagen with fibrillar- and fibril-associated supramolecular structures are the most common. Gelatin, the most common derivative is usually obtained by partial hydrolysis of collagen [87]. The presence of extensive crosslinking in mature collagen makes it difficult to dissolve in a wide range of solvents, which limits it processability [86]. However, the presence of crosslinks contributes to exceptional mechanical properties such as tensile strength and elasticity. The gelation process is thermoreversible. On cooling below 35 °C, crosslinking occurs via a transition of disorder to order as random coils return to an ordered triple helix state. It is therefore important for applications in physiological temperature ranges, that the gelatin is crosslinked to prevent its dissolution. Both collagen and gelatin have been widely used for biomedical applications such as drug delivery and tissue engineering [[88], [89], [90], [91], [92]]. They have been processed into different form factors such as films, sheets, sponges, hydrogels, and particles to facilitate their use [93]. As with many biological materials, enzymatic degradation by collagenase makes them suitable as substrates in transient electronics.

**Fig. 5:**
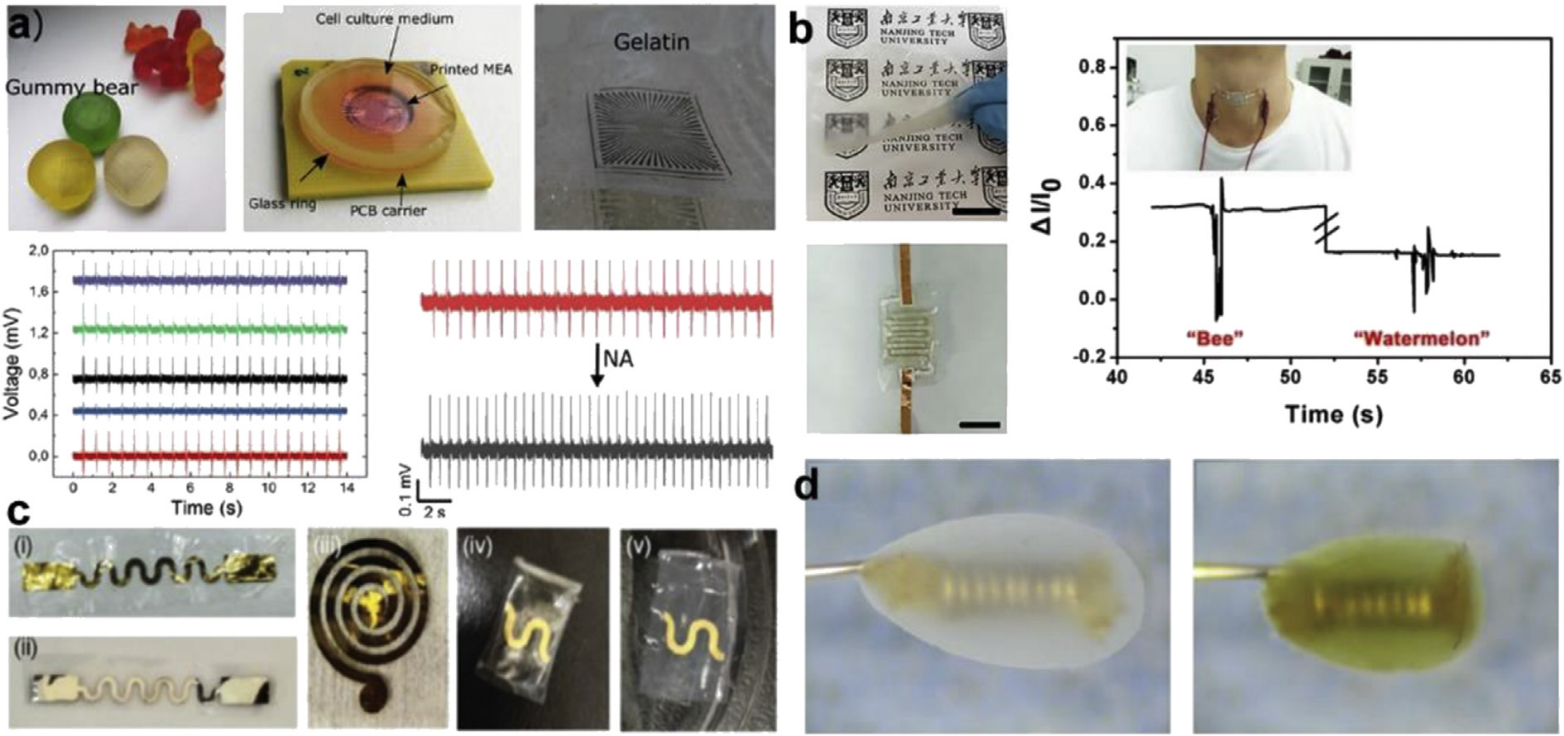
**Collagen-based devices**. **(a)** A carbon multi-electrode array (MEA) was printed on a gummy bear substrate (gelatin) for extracellular recording—showing a final chip bonded to printed circuit board with HL-1 cells culture. Action potential recording from HL-1 cells and stimulation with noradrenaline (NA) [191]. **(b)** Large-scale uniform and transparent collagen films (scale bar = 1.5 cm) with AgNWs as a flexible and transparent pressure sensor. Recorded current signals versus time as the volunteer pronounced ‘bee’ and ‘watermelon.’ The top inset shows a photograph of the sensor attached to the neck of the volunteer [95]; **(c)** i, iii) Cr/Au and ii) Mg conductive strips on collagen substrates, iv, v) encapsulated Cr/Au film between two collagen films: as prepared sample and after 2 h in PBS buffer, respectively [94]. **(d)** Implantable sensors coated with glutaraldehyde (GA)-crosslinked porous collagen scaffold and nordihydroguaiaretic acid (NDGA) crosslinked porous collagen scaffold [103]. (*All images used with permission*).

The properties of collagen and its derivatives have been observed to be suitable as substrates in flexible and biodegradable electronics. Some representative devices featuring collagen are shown in Fig. 5. Apart from biocompatibility, transparency and insolubility under physiological conditions enhancing stability, are notable advantages [94]. There have been demonstrations of functional devices based on collagen and/or gelatin in combination with conducting materials. Transparent films of acid-soluble collagen were used as a substrate for depositing metal electrodes via electron-beam evaporation and inkjet printing. A strain sensor, temperature sensor, wireless antenna, and space heater were demonstrated. Leather waste in the form of pickled skin was used as the source of collagen fibers [95]. A regenerated collagen film using ionic liquids was used as a substrate to fabricate a flexible and transparent pressure sensor. Two collagen films coated with AgNWs were used as active layers, painted with Ag paste as interdigitated electrodes. The sensor could convert mechanical pressure into an electrical signal. Different motion ranges of the human body, such as voice recognition, finger and wrist bending–releasing motions could be discriminated. Here, the properties of collagen were vital to the fabrication, including minimal mechanical property mismatch between the device and the body. The use for collagen for electronic skins can therefore be envisioned.

Collagen is well known for promoting cell proliferation. Combining with CPs can facilitate substrates that can provide electrical stimulation to cells for functions such as neural regeneration. Stimulation was shown to promote neural cell (PC12) differentiation by increasing neurite branching and length [96]. The adherence of cells on electrode surface affects signaling at the neural-electrode interface. An electrochemically active composite of gelatin was formed by incorporating it into PEDOT-tosylate (PEDOT (TOS)) films via vapor phase polymerization [97]. No significant change in electrochemical activity on inclusion of gelatin was noted. Cell growth was observed in PEDOT (TOS)-gelatin films, but not on PEDOT (TOS) films. Collagen-calcium phosphate composites have been combined with PEDOT:PSS to provide electrical stimulation to enhance bone tissue and mineral growth [98]. A type I collagen membrane was formed by electrochemical aggregation, followed by deposition of calcium phosphate and electrochemical deposition of PEDOT:PSS.

PPy is another conducting polymer that has been combined with collagen via *in situ* oxidative polymerization, along with freeze drying to form aerogels [99]. A maximum conductivity of 3.59 × 10^−4^ S/cm could be achieved using a combination of FeCl_3_ and anthraquinone-2-sulfonic acid sodium salt as the oxidant and dopant, respectively. PPy-chondroitin sulfate films were functionalized with collagen via covalent coupling [100]. This linking did not significantly affect the electrical conductivity of the native films. Expectedly, the composite film showed significantly higher cell numbers than those without. When electrical stimulation was provided to the cells, there was a clear increase in the extent of neurite networks and neurite length. Such demonstrations highlight the importance of incorporating bio-derived materials such as collagen in conductive electrodes for providing stimulation and neural interfacing.

There have been a few reports on collagen-based sensors. A peroxide sensor was formed using an electrospun hemoglobin--collagen microbelt modified electrode [101]. Hemoglobin replaced conventional peroxidase owing to a similar structure and catalytic activity toward peroxides. Gelatin can provide a stable matrix for the immobilization of enzymes such as urease [102]. A label-free urea sensor was reported using a screen-printed organic electrochemical transistor (OECT) on a PET substrate with a urease immobilized in gelatin hydrogel bridging the PEDOT:PSS gate electrode and channel. Loss of function is a common problem encountered by implanted devices because of factors such as protein adsorption, inflammation, fibrous encapsulation, loss of vasculature, biofouling, and other physiological responses. To address this issue, a crosslinked type I collagen scaffold was formed around an implantable glucose sensor to increase stability and biocompatibility of the sensors [103]. A cell-based sensor for the detection of pathogenic microorganisms and toxins was formed by immobilizing a B-cell hybridoma in a collagen matrix. Detection was achieved by quantitative analysis of cell viability *in situ* in the presence of pathogenic microorganisms or their toxins [104]. A tubister (biomimetic transistor inside a tube) was fabricated with a PEDOT:PSS/collagen three-dimensional (3D) microporous scaffold as the channel between Au/Kapton electrodes as source and drain. A highly coiled Pt mesh was used as the gate electrode. MDCKII cells were cultured *in situ* while maintaining continuous flow of medium. To demonstrate application for real-time toxicology monitoring, EGTA (ethylene glycol tetraacetic acid) detection was carried out. The effect of EGTA concentration on 3D-cultured devices was evaluated as a function of progressive disruption of the cell barrier [105].

## 3 Polysaccharides

### 3.1 Cellulose and paper

Cellulose is the most abundant polysaccharide found in nature. Plants are the primary source of cellulose, followed by marine animals, algae, fungi, and bacteria [106,107]. Attractive properties such as low cost, widespread availability, renewability, light weight, along with biocompatibility and biodegradability (or composability) have led to extensive investigations on cellulose-based technologies. Cellulose is a linear condensation polymer with a flat ribbon-like structure. The cellulose chain is made up of d-anhydroglucopyranose units linked together by β 1–4 glycosidic bonds [107]. Depending on the source, a cellulose polymer typically consists of ~400–10,000 repeating units. The high chain length along with extensive hydrogen bonding hinders easy processing. Cellulose is known to be insoluble in water and most common organic solvents. Nonetheless, efforts have led to the emergence of various processing techniques. *N*-methylmorpholine-*N*-oxide is a widely used solvent for dissolving cellulose [108]. LiCl/*N*,*N*-dimethylacetamide (LiCl/DMAc), aqueous NaOH solution, alkali/urea, and tetrabutylammonium fluoride/dimethyl sulfoxide (DMSO) are some other common solvents used. The application of ionic liquids for the dissolving cellulose has also been explored [[109], [110], [111], [112], [113]].

**Fig. 6:**
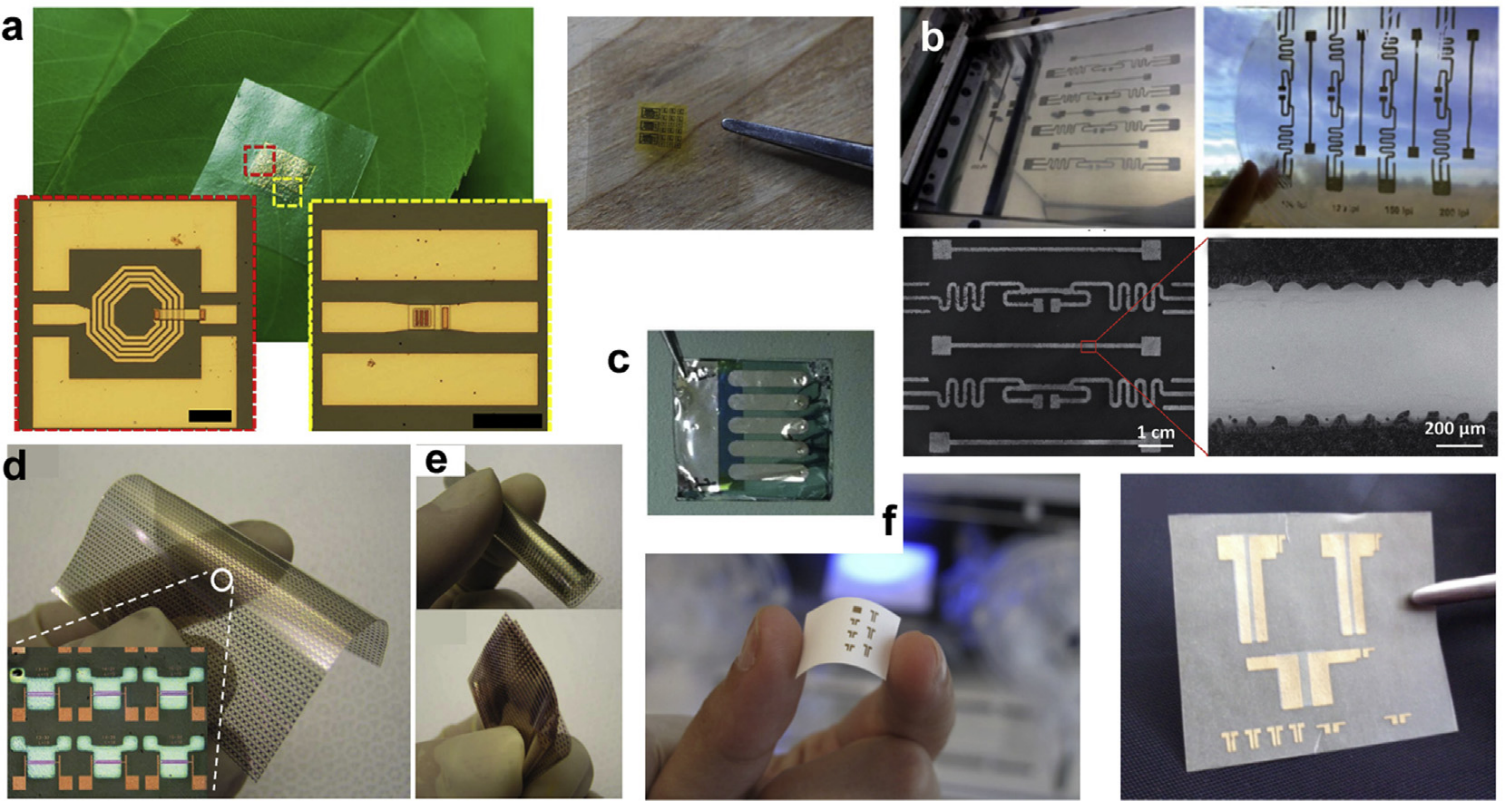
**Cellulose-based devices**. **(a)** Array of inductors and capacitors on a cellulose nanofibril (CNF) substrate put on a tree leaf, with optical image of the measured 4.5 turn inductor and metal–insulator–metal (MIM) capacitor. Scale bar = 100 µm. CNF paper with digital electronics [118]; **(b)** A gravure printed antenna on shape-stable transparent nanopaper with four radio-frequency identification (RFID) antennae and closeup of the antennae [119]. (**c**) Fabricated solar cells on CNC substrates using CNC/Ag/PEIE/PBDTTT-C:PCBM/MoO_3_/Ag [123]. (**d**) Photograph and optical microscopy image of 20-μm-thick transparent nanopaper-based OTFT array. Short-channel TFTs with a resolution of 70 × 70 were integrated in a 70 × 70 mm area. **(e)** The OTFT array in bending and folding states [120]. **(f)** Representation of an electronic device where the cellulose paper is simultaneously the gate dielectric material and the substrate. The CMOS using n-type and p-type metal oxide semiconductors [121]. (*All images used with permission*).

Cellulose is often converted to derivatives by modifying the H-bond network and introducing different substituents with desired properties. Such processing techniques have led to a multitude of cellulose-based organic electronics and biodevices [114]. Paper derived from cellulose has been of great interest in its own right, as a low-cost substrate for building electronic devices because of its light weight, low cost, environmental friendliness, and ease of fabrication. Paper-based microfluidics are finding use for various diagnostic devices [115]. Advances have been made to improve the performance of paper devices for applications, such as electronic components, energy storage, antennas, and electronic circuits [116,117]. Here, we focus on examples of devices wherein cellulose itself has been used as the structural or functional material. Cellulose is intrinsically non-conductive, but can serve as an effective and stable carrier, matrix or scaffold for other conductive materials to form conductive biocomposites. Some representative devices featuring cellulose are shown in Fig. 6.

Initially, we consider the use of cellulose as a flexible substrate, especially in printed electronics as a viable alternative to plastic. Cellulose nanofibrils (CNFs) are preferred because they are more flexible in comparison to cellulose nanocrystals that are brittle. Energy storage devices, organic solar cells (OSCs), optoelectronic devices, transistors, and organic light emitting diodes (OLEDs) have been fabricated on cellulose substrates [118]. The low porosity of the cellulose films presents an ideal platform for printing components such as radio-frequency identification (RFID) antennas as they reduce the chances of breaking the conductive layer [119]. Similarly, an OTFT on a CNF substrate has been reported [120]. The gate dielectric layer was spin-coated on the substrate and displayed a low leakage current attributed to the surface smoothness. No degradation in performance of the device was observed after 1 h of function under bent conditions.

A thin, transparent nanopaper-based field effect transistor (FET) was shown, where a cotton-based nanocrystalline cellulose was used as the substrate as well as dielectric layer [121]. The FET reported better performance in comparison to paper and oxide-based thin-film transistors. Supercapacitors have also been reported wherein the electrodes were fabricated using a transparent layer of carbon nanotubes (CNTs) on bacterial cellulose (BC) nanofiber substrates. An ionic gel was used as the electrolyte between the conductive electrodes [122]. With a view toward sustainability, OSCs have been reported on cellulose substrates. CNFs are preferred to produce films of low surface roughness [123]. The solar cell consisted of a modified Ag layer as a semitransparent bottom electrode. The top electrode was prepared by evaporating MoO_3_/Ag onto a photoactive layer. Although the solar cell displayed competitive open voltage current and fill factor, the power conversion efficiency is still low compared to glass/ITO-based solar cells.

In addition to its use as a substrate, cellulose has been considered as the active component via formation of composites with CPs, CNTs, graphene, metal oxides, and ionic liquids. Such composites are usually realized by blending, grafting, doping, or co-network with cellulose allowing them to be used as the active material. PPy, polyaniline (PANI), and PEDOT:PSS are some common conducting polymers that have been combined with cellulose to construct conductive films [[124], [125], [126], [127], [128], [129]]. Combining CPs with cellulose can help overcome their typical problem of brittleness. As many CPs can maintain their conductivity in room temperature, the composites have been used to realize humidity, temperature sensors, and gas sensors. A biodegradable and flexible nanocomposite based on cellulose and PPy was reported in separate studies. Nanoscale PPy was introduced onto the cellulose surface to design a flexible, biodegradable, disposable, and low-cost humidity and temperature sensor [130,131]. A stable and fast humidity sensor was reported using a PANI–carboxymethyl cellulose as the biopolymer composite synthesized by simple polymerization [132]. The impedance of the system increased with the change in humidity. PANI–carboxymethyl cellulose composite was used as a liquid petroleum gas sensor [133]. An ammonia gas sensor was reported using cellulose/TiO_2_/PANI as a nanocomposite electrode. TiO_2_ nanoparticles were adsorbed onto the surface of regenerated cellulose nanofibers by immersing the nanofibers into the TiO_2_ solution. This was followed by *in situ* polymerization of aniline using the cellulose/TiO_2_ composite nanofibers as templates [134].

Conductive cellulose biocomposites can provide a benign environment for the immobilization of biomolecules such as enzymes. An enzymatic biosensor on a composite of PPy nanoparticles and ethyl cellulose was used to immobilize glucose oxidase (GOx) [135]. A sensor based on GOx immobilized-tin oxide–cellulose nanocomposite showed a good linear response in the physiological range of glucose [136]. Cellulose membranes can also serve as protective barriers that can be fine-tuned to allow efficient diffusion of glucose, while retaining GOx [137]. It has been reported to slow down the diffusion of interferents such as ascorbate and acetaminophen, while permitting a steady diffusion of glucose and hydrogen peroxide [138]. Because of its biocompatibility, cellulose has also been used to immobilize cells. A cellulose–SWCNT/Au sensor was used to immobilize leukemia K562 cells on a gold electrode in a cell impedance sensor [139]. The cellulose–SWNT composite was prepared using a room temperature ionic liquid as the intermediate solvent and drop cast on Au electrodes. Sensors for leukemia K562 cells showed a linear range from 1.6 × 10^4^ to 1.0 × 10^7^ cells/mL, with a detection limit of 2.6 × 10^3^ cells/mL.

Conductive cellulose composites were used to form lightweight, environmentally friendly energy storage devices. A nanostructured high surface area electrode material composed of cellulose from algae coated with a thin layer of PPy was used as an energy storage device [140]. A conductive BC nanofiber/PANI composite with a conductivity of 5.1 S/cm was developed. Preparation was optimized to obtain the highest electrical conductivity with well-controlled morphologies. A high specific capacitance of 273 F/g was reported at 0.2 A/g current density [141]. A solid state and flexible aerogel supercapacitor based on a composite of CNFs, PANI, and Ag was reported with comparable specific capacitance.

Cellulose derivatives, such as carboxymethylcellulose, have been reported to have good SWNT dispersing abilities [142]. A highly sensitive, room temperature gas sensor was used for the detection of NO_2_ and NH_3_ using a cellulose–SWNT composite [143]. The sensor exhibited high sensitivity toward NO_2_ and NH_3_ with detection limits of 25 ppb and 5 ppm, respectively. Gallium nitride (GaN) is an emerging semiconductor material as a silicon replacement. GaN was used as a conductive coating to form conductive flexible cellulose paper [144]. Interdigitated gold electrodes were patterned to assemble an NH_3_ and NO_2_ gas sensor with fast response and recovery time. An acid sensor was reported with sheets of cellulosic paper modified with PANI nanoparticles [145]. When exposed to acidic conditions, strips of the modified paper sheets are subject to RGB color changes that can be detected by a scanner. A hybrid nanocomposite of TiO_2_, MWCNT, and cellulose was reported for the fabrication of a conductometric pH sensor. The large surface area of TiO_2-_coated MWCNTs improved the linear sensitivity of the pH sensor [146].

A multisource energy harvesting (mechanical and thermal sources) device was reported using a ZnO–cellulose nanocomposite [147]. Such devices can be scaled or stacked up to increase and enhance energy harvesting capabilities. A piezoelectric generator for mechanical energy harvesting was reported based on native cellulose microfibers [148]. The voltage generation of the device was assessed by imparting a stress of amplitude 40 kPa via human hand punching. The device showed an open circuit voltage of ~30 V corresponding to a power density of ~9 μW/cm^3^, which is sufficient to power several LEDs and small electronics. Such devices obtained from bio-derived materials can be used to generate power through cardiac motion, blood circulation, and other types of mechanical strain generated inside the body.

### 3.2 Chitin

Chitin is the second most abundant natural biopolymer in nature after cellulose. It forms the basic structural material in the exoskeleton of marine crustaceans and arthropods (e.g. crab, shrimp, lobster), but can be also found in some fungi, algae, and similar microorganisms [149,150]. A copolymer of glucosamine and *N*-acetyl glucosamine, chitin is a polysaccharide with a linear chain composed of (1–4)-linked 2-acetamido-2-deoxy-β-d-glucopyranose units, with a structure very similar to that of cellulose. Chitosan, which is a partially deacetylated form, is the most common derivative of chitin. Chitin and chitosan are of commercial importance as good chelating agents. Unlike several natural polysaccharides such as cellulose, dextran, pectin, carrageenan, and son on, which are mostly neutral or acidic, chitin and chitosan are basic. Properties such as biocompatibility, non-toxicity, biodegradability, low immunogenicity, antimicrobial properties, and ease of availability have increased their adoption for biomedical applications. Both chitin and chitosan have been widely used as passive materials for tissue scaffolds, drug delivery vehicles, antibacterial and antifungal agents, and wound healing [[151], [152], [153], [154], [155]]. The low reactivity and solubility make it difficult to process chitin in forms suitable for biodevice application. This is mainly due to strong intra- and intermolecular H-bonds resulting in a highly aggregated 3D network. Chitosan derived by deacetylation and depolymerization of native chitin is soluble in dilute acids, which has led to its popularity over chitin. A few commonly used solvents for chitin include 5–7% LiCl in DMAc and *N*-methyl-2-pyrrolidone (NMP) [156].

**Fig. 7:**
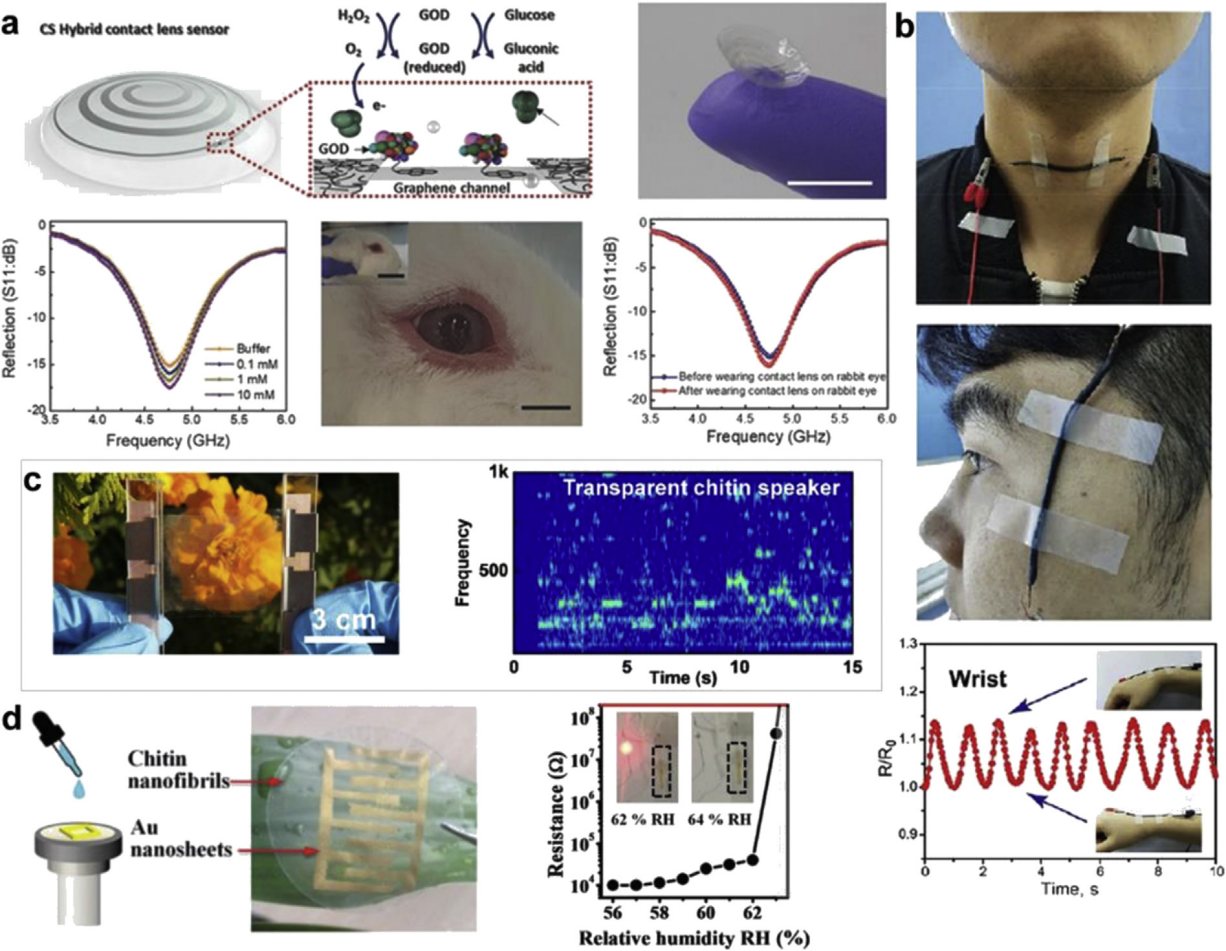
**Chitin-based devices**. **(a)** A chitin-silk hybrid-based contact lens sensor for wireless glucose monitoring showing schematic, monitoring of concentrations from 1 × 10^−3^ to 10 × 10^−3^ M and lens applied on a rabbit eye (scale bars = 1 cm). Wireless sensing curves of the contact lens sensor on a rabbit before and after wearing [162]. **(b)** A strain sensor based on spirally structured composites with carbon black, chitin nanocrystals, and natural rubber. Photograph showing the strain sensor attached to the throat and recorded current signals when the tester conducted a wrist movement [161]. (**c**) Transparent chitin speaker and microphone sandwiched between two AgNW electrodes with STFT signals verifying high-quality sound performance [159]. **(d)** flexible hybrid circuits produced from Au nanosheets for high humidity-sensitivity patterned on a film of ChNFs via sequential filtration. Humidity-sensing evaluation via the electric conductivity of the hybrid circuit. Transition from conductivity to insulation induced by enhancing the humidity from 62% to 64% [157]. (*All images used with permission*).

The fabrication of high-quality, mechanically strong chitin films make them suited as substrates in flexible biodevices. Some representative devices featuring chitin are shown in Fig. 7. A flexible green circuit was prepared on a chitin nanofibril film by exploiting the strong adhesion to Au surfaces [157]. This allowed the circuit to be used as an ultrafast and sensitive humidity sensor as a function of swelling of the substrate, wherein the swelling of the hydrophilic films destroyed the contacts between gold nanosheets. A chitin nanofiber (ChNF) paper was created using a solution of β-chitin in hexafluoroisopropanol (HFIP) and subjecting it to centrifugal casting to produce uniform transparent paper. β-Chitin was chosen over α-chitin because the former is readily soluble. To demonstrate its potential as substrate for green electronics, an OLED device was fabricated on the ChNF paper.

A 3D foldable nanopaper with high conductivity was reported using a ChNF-based hydrogel [158]. The conductivity of the paper was reported to be 4.5 S/cm, facilitating its use as an electrode and a current collector without a metallic support. The inherent piezoelectric property of chitin was harnessed to develop a flexible transducer [159]. The transparent chitin film was demonstrated as a speaker by functionalizing with AgNWs. Conductivity was imparted to chitin nanosheets via interfacial assembly, followed by carbonization in a pyrolysis chamber, resulting in graphene-like carbon nanosheets. The chitin-derived carbon nanosheets could be dispersed into various solvents, which were used as the active component in supercapacitors, flexible circuits, and strain sensors. When the chitin nanosheets and carbon nanosheets were mixed together to form a hybrid film, the reported conductivity was as high as 3.7 S/cm for films containing 90 wt% carbon nanosheets. The conducting layers were deposited on an elastic latex membrane to form a bilayer strain sensor [160].

Chitin has also been combined with other naturally derived materials to enhance functionality. A high-performance strain sensor was reported by solution mixing and spray drying layers of conducting carbon black on chitin nanocrystal (ChNC) and natural rubber substrates [161]. The conductive sheet displayed high tensile strength and high elasticity attributed to the ChNCs and rubber, respectively. Such devices can be developed as wearable strain sensors to detect flexural movement because of the high sensitivity and low detection limit of the sensor. A ChNF and SF biomimetic (CS) hybrid has been used to create large area and 3D curvilinear films [162]. By fabricating an FET, a CS hybrid contact lens–based wireless sensor for monitoring the glucose concentration in tear fluids of rabbits was demonstrated. The CS hybrid was used as a wearable heater and scratch-resistant transparent display.

Native chitin and chitosan are non-conductive and have been combined with metal oxides, conducting polymers, and carbon materials to make conducting hybrids [158,161,163,164]. Chitin has been blended with PANI in LiCl/DMA to form a conductive composite used to form free standing films. When these blends were doped with HCl, there was ~3 orders of magnitude increase in the conductivity of the films. The films were used as temperature and humidity sensors with reproducible response after numerous cycles. Chitin has been often combined with GOx via electrostatic immobilization to form thin films, or dispersed in carbon and/or metal pastes to form glucose sensors [[165], [166], [167]]. A glucose sensor was reported using a GOx-immobilized squid pen chitin film with deposited Au electrodes. A carbon paste electrode containing Pt powder and chitin powder with GOx was used as a glucose sensor for sports drinks [166]. Examples include a voltammetric glucose biosensor based on a chitosan-κ-carrageenan polyelectrolyte complex encapsulating GOx [168]. A herbicide (glyphosate [*N*-(phosphonomethyl)glycine]) sensor consisting of a carbon and/or tyrosinase conjugate immobilized in a chitosan matrix was used to form a screen-printed electrode. The amperometric sensor displayed good sensitivity in the nanomolar range and was able to function in water and soil samples [169]. Immunosensors for the detection of cancer, serotonin, histidine-tagged proteins, ochratoxin A, and monosodium glutamate have all been reported based on chitosan complexes [[170], [171], [172], [173], [174]].

Aqueous KOH/urea has been used to dissolve chitin and fabricate optically transparent chitin films of superior mechanical properties suitable for use as a flexible electronics substrate [175]. Techniques such as micromolding and microcontact printing have been shown to make micropatterned scaffolds [176,177]. A photocrosslinkable form of chitin using commercially available chitin from shrimp shells was reported to form patterns and device-grade films, thereby opening up routes to use chitin for various functional devices [178]. Such demonstrations provide new opportunities for chitin-based flexible and wearable electronics.

### 3.3 Polysaccharides derived from seaweed

#### 3.3.1 Alginate

**Fig. 8:**
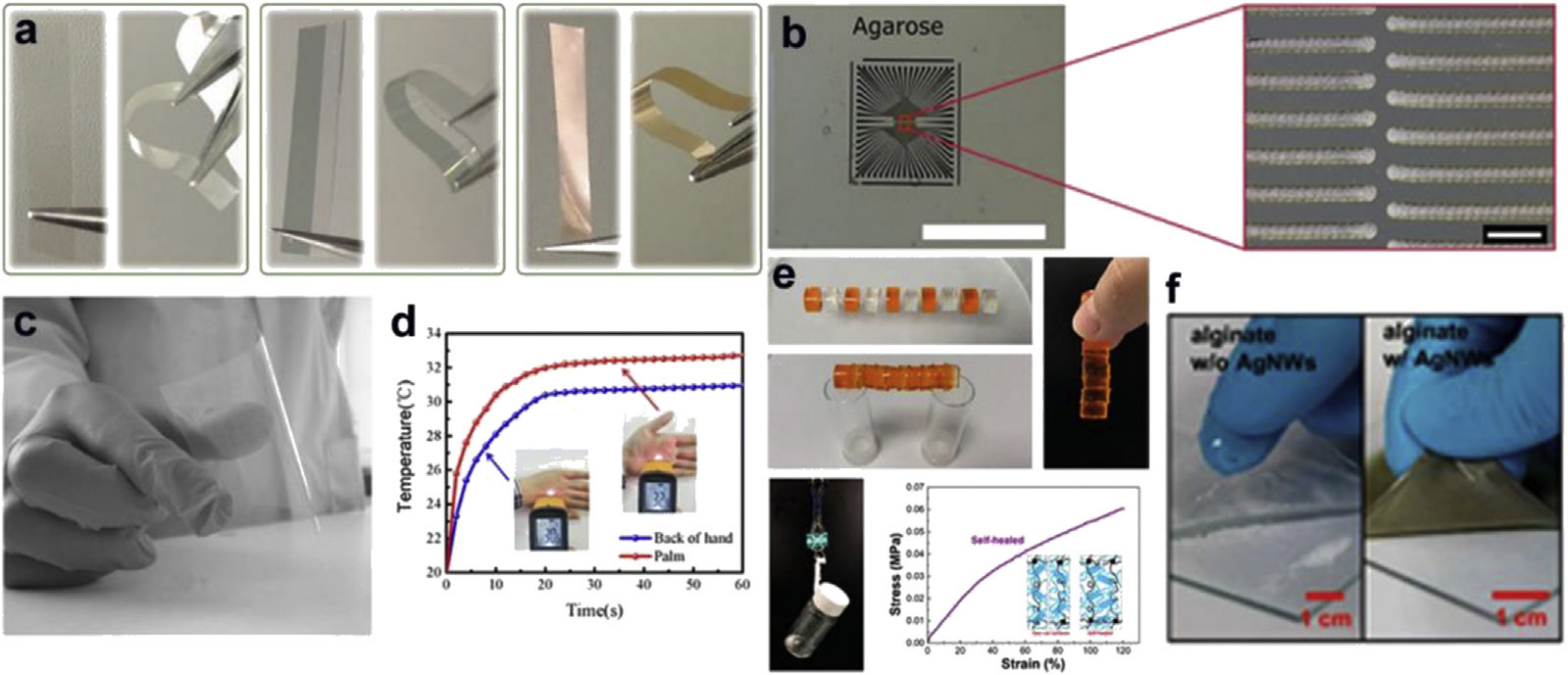
**Agarose/Alginate-based devices**. **(a)** Images of bare SA film, with 6 and 24 nm sputtered Au layers on top in straight and bent conditions [180]. **(b)** Image of printed MEA on an agarose substrate for extracellular recording (scale bar: 100 μm) [191]. **(c)** Printed films of gellan gum and carbon nanotube–gellan gum composite films on PET substrates [209]. **(d)** Flexible wearable graphene/alginate composite non-woven fabric temperature sensor monitoring the temperature difference between the palm and the back of the hand [182]. **(e)** Sensors based on spirally structured composites with carbon black, chitin nanocrystals, and natural rubber hydrogel blocks; five of them were strained using an orange-colored food dye. The cut hydrogel sample healed completely into one cylindrical block, and was able to construct a bridge. The self-healed dumbbell-shaped hydrogel sample with a cross-sectional size of 2 × 5 mm could hold a reagent bottle of over 15 g with tensile stress–strain curve [161]. **(f)** Nanocomposite alginate hydrogels with and without AgNWs [183]. (*All images used with permission*).

Alginate, or alginic acid, is a linear polysaccharide derived from the cell walls of brown algae species such as *Laminaria*. It contains covalently linked blocks of (1,4)-β-d-mannuronic acid (M) and α-l-guluronic acid (G) in similar or alternating patterns. A greater G:M ratio leads to higher stiffness and less porosity. Alginate strands can be ionically or covalently crosslinked, and the mechanical properties of elasticity and stiffness can be tuned by varying the G:M ratio, as well as the concentration of ions or covalent crosslinkers. Alginates are biocompatible and biologically inert, with hydrophilicity leading to poor cell adhesion, and they do not undergo enzymatic degradation [179]. Alginate hydrogels are degradable via alginases and dissolve slowly at neutral pH. They have previously been explored for wound healing, but recent work has explored their potential for electronic devices. Their properties can make them good candidates for biosensors because they offer a stable platform for encapsulated cells or other components. Some archetypical device examples are shown in Fig. 8.

Sodium alginate (SA) has been used to form a substrate for flexible conducting films. SA films (20 μm thickness) were produced via solvent-casting, then coated with a smooth ultrathin Au layer (<10 nm) via sputtering. The conductive properties of the film were comparable to ITO. At a thickness greater than 4 nm, the resistance exponentially decreased. The foils could be dissolved in water, and Au could be recovered via centrifugation. The films were stable for months at ambient conditions [180]. Alginates can also be blended with conductive materials to form composites that take advantage of ionic crosslinking properties. Electrodes for supercapacitors have been made using SA as an additive with higher specific surface areas to improve performance. Higher SA resulted in increased micropores and surface grooves, as well as fiber thickness. When used in mats for electrical double-layer charging, the capacitance was increased [181].

Flexible resistor-type temperature sensors that can adhere and conform to human skin have used alginate composites as sensing components. SA was used as a graphene nanoplate dispersant, then converted to calcium alginate fibers via wet-spinning in a CaCl_2_ bath. Binding of randomly arranged fibers was used to create a non-woven fabric. The resistance decreased with increasing temperature after considering the effects of strain and relative humidity. The device had a rapid response time, and exhibited good stability and cyclability [182]. Stretchable, soft, conductive nanocomposites using alginate hydrogels and conductive AgNWs have also been developed for wearable electronics. Alginate/AgNW hydrogels were ionically crosslinked using CaCl_2_, then a layer of polyacrylamide (PAAm) hydrogel was attached underneath as a stress-releasing layer to match the mechanical properties of human skin. This material could be laser-cut, and was used to form a coil-shaped electrode for a skin-mountable antenna, with the active layer and current collector on a single layer. AgNW–V_2_O_5_ solution was mixed with alginate and AgNW to form two supercapacitor layers, sandwiched with a hydrogel electrolyte layer of PAAm and 1M Na_2_SO_4_. When bent on skin, there was negligible change in capacitance [183].

#### 3.3.2 Agarose

Agarose is a linear polysaccharide obtained from red seaweed, and is one of the two main units that makes up agar. Agarose is purified from agar by removing the second component agaropectin. The agarose polymer chain consists of alternating d-galactose and 3,6-anhydro-l-galactopyranose linked by α-(1 → 3) and β-(1 → 4) glycosidic bonds [184]. Agarose has been used since the 17th century as a gelling agent. It is a thermoreversible ion-dependent gelling agent and exhibits thermal hysteresis in its liquid-to-gel transition [185]. Agarose is obtained as a white powder soluble in boiling water, but not in cold water. It is also readily soluble in polar aprotic solvents, such as DMSO, dimethylformamide (DMF), formamide (FA), *N*-methylformamide (MFA), and ionic liquids [186]. Agarose has been used as a growth medium for bacteria and fungi, as a macromolecular support for gel electrophoresis, and for immobilization of cells and enzymes [184]. As a biocompatible material, agarose and its derivatives have been widely used for tissue engineering applications [186]. Agarose can be emulsified and cast on different substrates to obtain films, discs, hydrogels, and microspheres [[187], [188], [189]]. These unique properties have accounted for some attempts to use it for biodevices. Some archetypical device examples are shown in Fig. 8.

Agarose has been used as a biodegradable, transparent, and flexible substrate on which a current collector ink was stencil printed followed by the printing of two electrodes using Ag_2_O ink and Zn ink. An antibody and aluminum were also printed on the agarose substrate to demonstrate its compatibility with lithographic processes for use in flexible antennas and disposable biosensors [190]. Microelectrode arrays were printed on an agarose hydrogel substrate using ink jet printing, showing its compatibility with different lithographic processes [191]. A carbon nanoparticle ink was directly printed on agarose followed by the passivation layer. The reported microelectrode arrays were applied for the localized recording of action potentials from HL-1 cells demonstrating their utility for electrophysiological measurements.

To form conductive composites, agarose has been combined with materials such as PPy, CNTs, GOs, and AgNW. In this regard, the agarose hydrogel is often used because of the reversible sol-gel transition, which allows the dispersion of a hydrophilic filler, giving it a high degree of moldability. A humidity sensor was formed using an agarose conductive composite microfiber with rGO and AgNWs [192]. The synergistic effect of the two conductive fillers resulted in a higher conductivity. The absorption of water on the GO defect sites increased the conductivity of the composite, which enabled its use as a humidity sensor. A humidity sensor was also reported using a composite made from CNT fillers in an agarose hydrogel matrix. Sensing was achieved as a function of increase in resistance because of the absorption of water, initiated by the swelling of the hydrogel in the presence of moisture [193]. PEDOT/agarose electrodes have been prepared by electropolymerization on a melted agarose hydrogel poured over Pt electrodes followed by peeling off using electrochemical actuation. The electrode was used to stimulate C2C12 myotubes, with the electrical stimulation inducing a contraction of the muscle cells [194]. Furthermore, an NaCl-doped agarose gel was used as the biocompatible conductive filler inside a 3D-printed elastomer matrix to fabricate a wearable sensor with long-range stretching sensitivity. The NaCl-doped gel placed in a 3D spring conformation displayed more sensitivity toward bending, whereas the same gel placed in a 1D conformation had a dominant stretching signal. When both conformations are integrated, a motion sensor with high selectivity toward bending and stretching was realized [195].

Agarose hydrogels are also useful in biocompatible and biodegradable energy storage devices as degradable electrolytes in microsupercapacitors (μSCs). A fully biodegradable μSC using transient metals, such as W, Fe, and Mo electrodes, was fabricated on a biodegradable poly (lactic-*co*-glycolic acid) substrate with an NaCl/agarose gel electrolyte [196]. The hydrogel electrolyte induced the formation of metal oxides with each charge/discharge cycle, thus increasing the pseudocapacitive behavior leading to a higher performance. A μSC based on PEDOT:PSS used a silk sericin carrier printed on an SF substrate with NaCl/agarose gel as the benign electrolyte. The μSC was shown to be cytocompatible and possessed tunable enzymatic degradability ranging to a few weeks [197]. Such demonstrations where multiple bioderived materials are used for the realization of devices are particularly interesting. Agarose was used in the fabrication of the electrodes as a CNT-embedded agarose composite fiber for the fabrication of a flexible and wearable μSC. The μSC was coated with PDMS to impart water resistance and stability [198]. Agarose has been used as a binder material in high-performance rechargeable Li-ion batteries by promoting a strong adhesion between the Si active material and copper current collectors. The agarose was also used as a source for hard carbon, which was combined with Si to form the active material [91].

### 3.4 Other polysaccharides

Gellan gum (GG) is a microbial polysaccharide produced by the fermentation of *Pseudomonas elodea*, a gram-negative, non-pathogenic bacterium [199]. The GG chain consists of repeating units of α-l-rhamnose, β-d-glucose, and β-d-glucuronate, in the molar ratios 1:2:1 [200]. In the solution state, the molecules undergo a transformation from the disordered state (single chain) to the ordered state (double helix) with decreasing temperature and above a certain critical concentration the double helices form aggregates [201]. Currently, GG is approved for food additive use as a gelatin replacement. It has been extensively studied for pharmaceutical formulations, dental care, tissue engineering, bone repair, and drug delivery [[202], [203], [204], [205], [206], [207]]. Although there have been a few examples of GG-based biodevices, poor mechanical properties, instability in physiological conditions, and high gelling temperatures make it a challenging candidate for device applications.

A GG-graft-PANI was reported in which PANI radicals were formed by persulfate-stimulated oxidative polymerization of aniline. They were mixed to form GG-graft-PANI graft copolymer for use in electrochemical sensors, transistors, energy storage devices, and neural interfacing devices [208]. As GG is a well-known biopolymer dispersant, it has been used to disperse and stabilize CNTs in an aqueous solution ink jet printed on PET substrates [209]. A GG hydrogel was formed containing CNTs, wherein the conducting hydrogels were used to stimulate incorporated cells toward functional tissue formations [210]. A conductive hydrogel using GG and PANI was reported where prolonged stability and promotion of adhesion, proliferation, and differentiation of myoblasts into myotubes was shown [211].

A combination of GG with hemoglobin and an ionic liquid (BMIMPF6) was used for the fabrication of a hydrogen peroxide sensor. The conducting gel composite was applied for the surface modification of glassy carbon electrodes. Peroxide detection was performed electrochemically by measuring the electrocatalytic reduction peak current [212]. A stretchable and self-healing strain sensor was reported using GG and polyacrylamide. The recoverability and self-healing properties of the hybrid hydrogel can be attributed to the thermoreversible nature of GG gelation. No hysteresis was observed in conductivity measurements during stretching and the strain sensor was able to detect tiny physiological changes, demonstrating its sensitivity and potential as wearable electronic sensors [213].

Another widely available, low cost, polymeric carbohydrate is starch that is primarily used for energy storage in plants. Starch consists of numerous glucose units joined by glycosidic bonds. Starch from potatoes and chitosan from crab shells were combined in a nacre-inspired 3D interconnected single-wall CNT–pristine graphene (PG)–PEDOT:PSS network architecture as a transparent electrode. The formation of the conductive network led to a low sheet resistance and superior flexibility. The edible starch–chitosan substrates could be biodegraded in lysozyme solution rapidly at room temperature [214]. A similar material using potato starch–chitosan composite was used to form a robust, non-volatile, flexible, and transparent resistive switching memory (ReRAM) device [215]. Starch is a particularly interesting biopolymer in the discussion of nature-derived electronics because of its use in Ecoflex™, a commercially available and compostable flexible polymer, certified by BASF. Ecoflex™ is blended from corn starch, potato, and polylactic acid, and has been used in various devices including a deformable resistive temperature sensor. The magnesium, silicon dioxide, and nitride microstructures were encapsulated with ultra-thin films of Ecoflex™. This entire structure is potentially compostable [216].

## 4 DNA

We briefly discuss the use of nucleobases (primarily deoxyribonucleic acid [DNA]) in devices, as they fall under the broader umbrella of natural materials. The use of DNA is somewhat different from the other natural materials because of its complexity and use, not just as a structural material, but wherein its unique properties and engineered architectures lead to different applications. We therefore restrict our discussion to a few examples to display this potential. DNA is a sophisticated, self-replicating material, which is the carrier of genetic information in most living organisms. By the virtue of complementary base pairing, DNA has attractive properties such as specific molecular recognition and the ability to form supramolecular structures through self-assembly [217]. Various studies have shown that DNA can behave as an electrical conductor [218,219]. With proper connections to a suitable donor and acceptor, DNA can facilitate charge transfer over long distances [218]. DNA is a highly polar molecule because of the presence of a highly charged phosphate backbone, making it soluble and stable in aqueous environments, but insoluble in organic solvents [220]. However, they can be made compatible with organic solvents by modification with hydrophobic groups [221]. The combination of all these properties generates a potential for a host of DNA-based electronics.

DNA and ribonucleic acid (RNA) can be formed into 2D and 3D architectures of amazing complexity using various self-assembly techniques including crossovers, junctions, and origami [[222], [223], [224], [225], [226]]. These ideas have led to the development of various DNA nanotechnologies, which will not be covered here [227]. However, the precise placement of various elements in complex configurations in these architectures have created DNA-based devices such as sensors [228]. Sensors using specific molecular interactions for the detection of mRNA, DNA hybridization, single-nucleotide polymorphisms, as well as protein biomarkers, metal ions, and pH have been reported [[229], [230], [231], [232]]. A DNA origami capsule containing a signaling molecule was used as a malaria detecting system, where the capsules open and release a signal only in the presence of *Plasmodium falciparum lactate dehydrogenase* [233].

In terms of other conventional devices, DNA-based biopolymers have been used as the dielectric gate layer in organic field-effect transistors (OFETs) as an alternative to polymers such as parylene C and poly (methylmethacrylate) [234]. Purified DNA from marine salmon was transformed into a DNA--lipid complex by precipitation in water with a cationic surfactant complex, hexadecyltrimethylammonium chloride (CTMA) [235]. This made the DNA--lipid complex insoluble in water, but soluble in organic solvents. Apart from a gate dielectric layer, DNA was used as a buffer layer to enhance charge injection and carried mobility of OTFTs. An OFET device was reported with a spray-coated water-soluble DNA as the buffer layer between pentacene and electrodes [236]. The inclusion of the water-soluble DNA increased the field effect mobility, which was attributed to the elimination of bulk-like phase change of pentacene, and decrease of the high trap density through incorporating DNA in water.

In comparison to transistors used to regulate current flow using a gate voltage, rectifiers are used to channel current flow in a forward bias direction. A DNA-based molecular rectifier was shown by intercalating coralyne molecules into a specifically designed duplex DNA [237]. DNA and its derivatives have been used in a wide range of high-density memory devices such as write-once-read-many-times (WORM), reproducible write–(read)_*n*_–erase, and multilevel biomemristor, and for recording cellular events [[238], [239], [240]]. There have also been demonstrations of DNA in photovoltaic devices because of its electron extracting characteristics [241]. The DNA layer was placed between ITO and PTB7:PC_70_BM donor acceptor layer together with an MoO_3_ hole-transport layer. A DNA-based hybrid material in polymer solar cells was shown where the DNA:CTMA complex works as an optical absorption dilutor, while PEDOT-S provides the conducting pathway for electron transport [242]. A perovskite solar cell was reported which utilized DNA-CTMA complex as the hole transport layer. The DNA--CTMA layer was spin coated on FTO and thermally annealed at 80 °C. Interestingly, DNA–CTMA-based devices were reported to possess better life time in comparison to PEDOT: PSS based devices, with degradation starting after 10 days. These demonstrations highlight the role for DNA-nanotechnology in sensors, photonics, as interfaces between technical systems and living organisms, or for biomimetic fabrication processes [243].

## 5 Nature-derived active materials

**Fig. 9:**
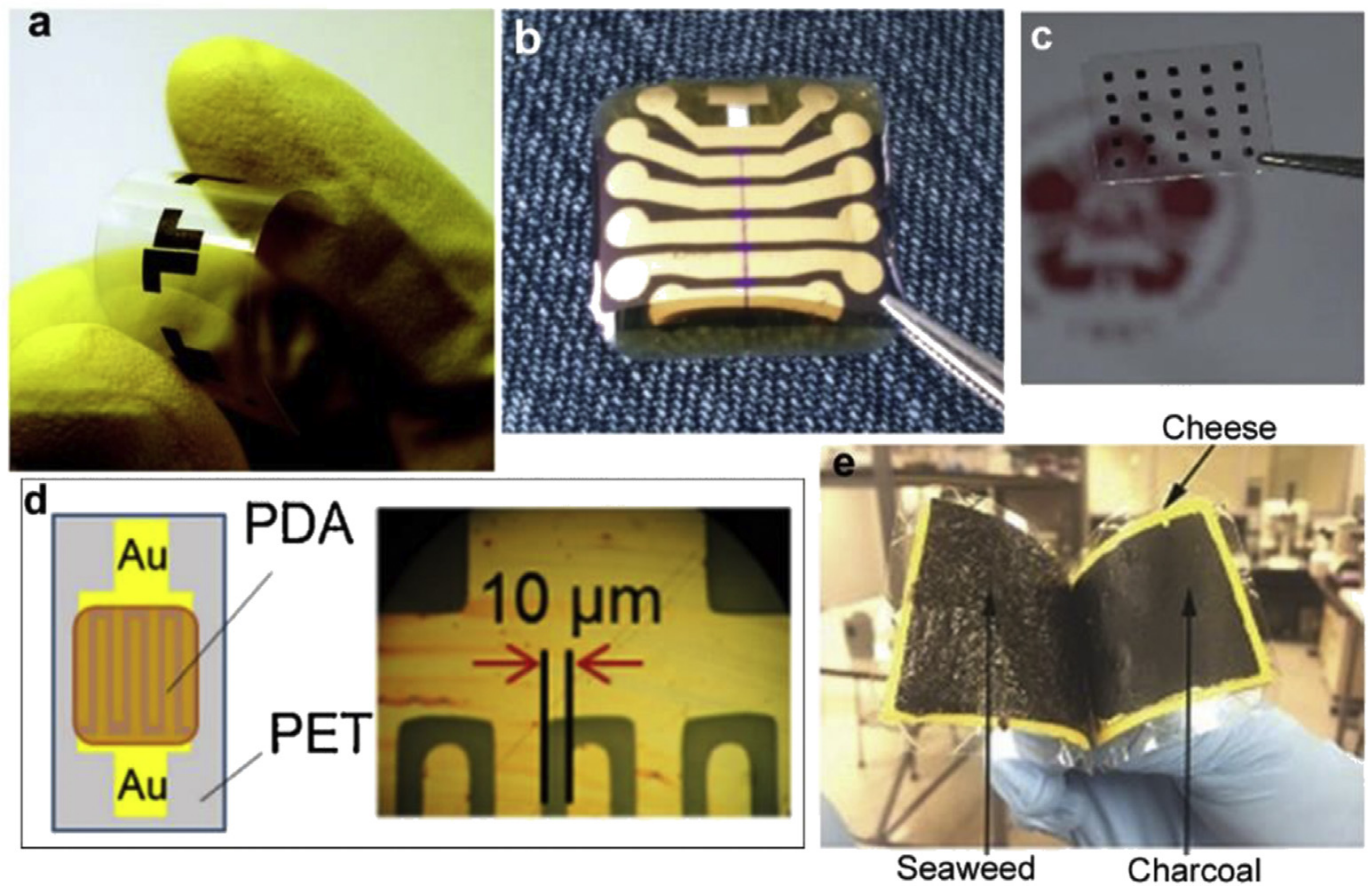
**Active and miscellaneous natural materials**. **(a)** Melanin-based flexible microsupercapacitors on a flexible PET substrate using NH_4_CH_3_COO as the electrolyte [258]. **(b)** Ambipolar indigo field-effect transistors fabricated on shellac with aluminum oxide–tetratetracontane dielectric and gold source and drain electrodes [264]. **(c)** Schematic illustration and image of a chicken albumen biomemristor printed on a glass substrate [278]. **(d)** A humidity sensor fabricated using a dopamine–melanin thin-film on gold electrodes [256]. **(e)** An edible supercapacitor showing the activated charcoal electrode, seaweed separator, cheese segregation layer, and gelatin package [282]. (*All images used with permission*).

In the above sections, the focus has been the use of nature-derived materials that primarily serve as the structural components of the devices—either as the pure material or as a composite. As noted, many are insulators or dielectrics, with conducting composites formed with organic and inorganic dopants, including metals, nanotubes, and conducting polymers. Nature also provides a host of active materials as we discuss below. These can provide electrochemical or optoelectronic behavior without the need to resort to synthetic alternatives. Although there are numerous organic dyes and pigments that are natural semiconductors, much of the work in this novel field has focused on understanding the fundamental properties of these materials. The relatively few examples that have been successfully used in or as devices have been discussed here. Some examples of reported devices are shown in Fig. 9.

### 5.1 Melanin

Melanins are a broad class of pigments found in a number of organisms. The human body contains three types of melanins, namely eumelanin, phenomelanin, and neuromelanin. The first two act as photoprotectants, whereas neuromelanin is produced in the brain. Eumelanin has been of interest because of its unique physical and chemical properties, such as broad band absorbance, free radical scavenging, as well as photo and electrical conductivity [244,245]. Interest in melanin was sparked because of its discovery as an organic amorphous semiconductor material [246]. Ever since, numerous attempts have been made to understand the exact mechanism of conduction [247,248]. Hydration-mediated proton transport is assumed to be one of the main processes that imparts conductivity [249,250]. Melanin undergoes a ‘switching’ behavior in the hydrated state, with a decreasing trend in conductivity observed with an increase in temperature [246]. Comprehensive models of charge transport in melanin are still being elucidated [248].

A major challenge is the limited solubility of melanin in most common organic solvents, which hinders its processing into form factors suitable for device fabrication [251]. Solution processing using solvents such as DMSO, DMF, and aqueous ammonia have been reported to obtain smooth films [247,[252], [253], [254]]. Melanin thin films processed from DMSO were shown to exhibit molecular scale smoothness and large area uniformity [252]. The nanoscale films with a conductivity of ~7 × 10^−5^ S/cm at 100% humidity. *In vitro* evaluation showed an enhancement of Schwann growth and neurite extension, whereas *in vivo* tests revealed an inflammatory response similar to that of silicone implants. NH_4_OH was used to solubilize synthetic and natural melanin to produce smooth device quality films [253]. Films free from metal ion contamination showed a conductivity of 2.5 × 10^−5^ S/cm at a relative humidity of 100%. An electrochemical method for the preparation of metal-supported electroactive melanin films was reported where the growth of eumelanin films was carried out from a solution in 0.1M NaOH under potentiostatic conditions [255].

The effect of humidity on the conductivity of melanin has been exploited for the realization of humidity sensors. A sensitive and rapid response sensor was reported using self-assembled dopamine–melanin thin films [256,257]. Oxidative polymerization of dopamine was carried out to form insoluble melanin aggregates in the presence of PANI, which acted as a deprotonating agent. Dopamine–melanin films were mounted onto a gold substrate to fabricate a sensor displaying good dynamic response to moisture. At any relative humidity, the thickness of the films showed a significant effect on the resistance of the films. The sensor was also used to assess the spatial distribution of humidity on fingers to function as a touchless position interface. A water-soluble dopamine–melanin could be synthesized through self-oxidation induced polymerization under basic conditions. Using solution casting, a dopamine–melanin thin-film sensor could be formed on interdigitated Au electrodes sputter-coated on a PET substrate. This resulted in an ultrafast humidity sensor with five orders of magnitude change in conductivity between 0% and 100% relative humidity.

Melanin has been used as an electrode material for pseudocapacitive energy storage systems using carbon paper as the current collector [258]. In addition, applications of melanin active layers in OLEDs, OECTs, OPVs, and OFETs have been explored because of its signal amplification capabilities at biological interfaces [259]. An OECT device was reported where melanin was used as an ion to electron transducer [260]. The choice of melanin stems from its ability to act as a mixed conductor, supporting both ionic and electronic currents. Vacuum thermal deposited Au on a glass substrate was used as the gate electrode, and solution-casted PEDOT:PSS was used as the channel. The transconductance of the PEDOT:PSS channel was modulated by the melanin. The OECTs were tested under ambient conditions, which provided adequate natural moisture to make the melanin conducting by facilitating proton percolation. An extended-gate FET was reported using a eumelanin film on a conductive substrate [261]. The conductive substrate (Au or ITO) was directly connected to a metal oxide semiconductor FET (MOSFET). The device was used for pH measurements. It was observed that the source-drain current increased with the decrease in pH of the solution, which can be explained by the diffusion of protons from the electrolyte layer, thus altering the conductivity of the melanin films.

### 5.2 Indigo

Indigo is one of the most well-known dyes in the world. Historically indigo was considered as a luxury item primarily used as a pigment for painting, but also for medicinal and cosmetic purposes. Traditionally, indigo has been obtained from the *Indigofera* genus in the tropical regions and *Isatis tinctoria* (the woad plant) in Europe. In plants, the precursor molecule is indoxyl, which is attached to the glucose ring forming glucoside indican. Indigo was produced synthetically from *o*-nitrobenzaldehyde and acetone on addition of dilute sodium hydroxide, barium hydroxide, or ammonia. Today, it is one of the highest produced dyes in the world commonly used to color cotton jeans. Chemically, indigo has extremely low solubility (only soluble in small amounts in DMSO, DMF, and similar polar protic solvents) and a high melting point of 390–392 °C, because of stabilization from the inter- and intramolecular hydrogen bonding and π–π stacking [262]. This limits its use and further derivatization. Often it is converted to a soluble latent pigment to allow easy processing. Multiple reports on the use of indigo and similar materials as a natural semiconductor have been published [[262], [263], [264], [265]]. It is speculated that the strong intermolecular interactions are also responsible for good charge transport properties observed in indigo. Moreover, the H-bonds and π–π stacking are responsible for ultrafast proton transfer in the excited state [262].

Such unique electrochemical properties have led to interesting demonstrations of indigo-based electronic devices. An indigo-based OFET was fabricated on a natural shellac resin [264]. The electrochemical properties of the indigo thin films were studied, and characteristic two-electron reduction and oxidation peaks were observed. For the OFET, aluminum oxide formed by the anodization of aluminum on the substrate was used as the gate. The gate was passivated by a thin layer of tetratetracontane (TTC), which is also a biodegradable polymer found in nature, as indigo shows semiconductor behavior on TTC. Although the OFET showed minimal degradation when stored in inert conditions, it degraded on exposure to air, which was attributed to the inability to transport electrons under oxygenated environments. A WORM memory device using a thin layer of indigo sandwiched between ITO cathode and Al anode was reported [266]. The interfacial dipoles formed between metals and organic materials were exploited. The system undergoes a transition on passing current. Before the transition, the injection barrier of holes from Al to the highest occupied molecular orbital of indigo is lower, which allows the device to be in a low resistance ON state. On passing of current, the dipoles are neutralized, which increases the injection barrier, thus transforming the device into an OFF state.

### 5.3 Carotenoids

Carotenoids are synthesized by certain plants and bacteria, and are responsible for some of the most beautiful colors present in nature, such as those in fruits and vegetables, leaves, and in some birds and animals. More than 600 different types of carotenoids occur naturally [267]. Some of the functions include light harvesting for photosynthesis, pigmentation, oxidative stabilization, and photoprotection. β-Carotene is the most commonly known from the carotenoid family, responsible for the color present in carrots, and can be extracted from tomatoes and carrots at low cost. As most of the extraction includes liquid processing, they are usually water soluble. The structure of β-carotene is similar to that of conducting polymers, with delocalized δ-electron systems [268]. The electrical conductivity of carotenoids is due to the presence of conjugated double bonds [269]. These electronic states that are delocalized over a chain of carbon atoms are also responsible for unique optical and semiconducting properties.

A field-effect semiconductor based on a range of different carotenoids, such as β-carotene, bixin, astacene, torularhodin, and isorenieratene, was tested [270]. The carotenoids were dissolved in THF and spin coated or drop casted onto test substrates. β-Carotene and bixin were reported to be p-type field-effect semiconductors. OFETs were fabricated using combinations with bio-derived materials to realize different device components [271]. Solution processed p-type β-carotene was used in this work to obtain an OFET. Glucose and caffeine were used as the dielectric layer. Inspired from the roles played by carotenoids in nature, a carotenoid-based OSC was reported. Three different solution-processed carotenoids, such as fucoxanthin, β-carotene, and lycopene, were combined with fullerene-derived PC_61_BM to form a bulk heterojunction OSC. The active layer of the OSC consisted of carotenoids as the electron donor and the PC_61_BM as the acceptor, between a MoO_3_-modified ITO substrate and Ca/Al electrodes. In another report, a β-carotene and PC_71_BM-based bulk heterojunction OSC was presented [272]. ZnO/PEDOT:PSS deposited on ITO was used as the substrate on which a composite of β-carotene and PC_71_BM in chlorobenzene or chloroform was used as the active layer.

## 6 Miscellaneous active and substrate materials

Nature presents an amazing diversity of materials (including several yet to be found) that display promise as substrates or active components of devices. Here, we list a few other materials of recent interest including the use of plant and animal wastes, food constituents, and so on. For instance, waste generated from citrus processing can provide a source of pectin, a complex carbohydrate polymer of α-galacturonic acid with variable methyl ester groups. Pectin from orange peels was used as an insulator to fabricate an organic resistive switching memory device with an Ag/Pectin/FTO structure. The device had good switching endurance accompanied by an OFF/ON resistance ratio of ~450 [273]. Extracted, and processed natural *Aloe vera* has been used as an active layer for memory applications. A functional memory device is fabricated using a bottom-up structure of ITO/*Aloe vera*/Al. From the current density–voltage measurements, the device exhibits a reproducible bipolar switching. Charges are transported across the *Aloe vera* layer via space-charge-limited conduction, and clusters of interstitial space formed by the functional groups of acemannans and de-esterified pectins in the dried *Aloe vera* contribute to the memory effect [274]. *Aloe vera* was also used to design an n-type OFET. A dielectric layer consisting of a mixture of extracted paste from fresh leaves with SiO_2_ nanoparticles and semiconductor, C60 was used [275]. Resins (plant and animal) and shellac (an amber colored substance secreted by an insect from the insect species *Tachardia lacca*) have been used in devices as substrate materials [276].

Some redox proteins also contain multiple charge-trapping sites in their 3D structures with reversible resistive switching making them good candidates for resistive random access memory (RRAM) devices and FET memories. Several researchers have reported on use of the tobacco mosaic virus, sugars, cysteine, ferritin protein, and various enzymes to fabricate resistive switching memory devices [277]. Chicken egg albumen-based biomemristors were fabricated by using the albumen as an insulator. The device comprised a metal (Al)/insulator (albumen)/metal (ITO) sandwich structure with significant bipolar resistive switching behavior. The switching property of the devices was remarkably improved by heat-denaturation of the protein [278]. Interesting nature-derived functional proteins include reflectin, found in a species of bobtail squid, *Euprymna scolopes*, and other cephalopods. Various cephalopod-derived biopolymers (e.g. eumelanins, chitosans, and reflectins) have been used in voltage-gated devices, such as transistors [279]. Reflectin films provide a means to mimic natural photonic structures and form a material for light transmission to be used in optical bioelectronics [280]. Other natural semiconductor materials include indanthrene yellow G and indanthrene brilliant orange RF [281].

Finally, there has been interest on moving toward fully organic and benign devices that use no, or minimal metallic components. An example includes a reported electrochemically functional, edible supercapacitors explicitly originated from edible and nontoxic food products, including activated charcoal, seaweed, cheese, and polyelectrolyte drink. The edible supercapacitors are demonstrated to kill disease-causing bacteria *in vitro* and to power a commercial snake camera [282]. The recently growing field of edible, absorbable and digestible electronics is of outstanding interest to interrogate biosignals within the body (specifically the gastrointestinal tract) and transform healthcare [283,284]. The use of naturally derived materials provides the perfect platform to advance such technologies.

## 7 Some perspectives on the use of natural materials

### 7.1 Sourcing from the environment

As noted above, the shift to natural materials is motivated by their typically low cost and widespread availability from a variety of flora and fauna. For instance, silk proteins are largely sourced from the cocoons of *B. mori* silkworms, a domesticated species that exclusively eats mulberry leaves, farmed extensively in Asia. While macroscale silk textiles can be energy-intensive, as it takes roughly 5000 silkworms to make a kilogram of silk, devices only require micrograms. Keratin can be sourced from hair, hooves, feathers, wool, and beaks that are the byproducts of industries such as tanneries or poultry. As keratin can be collected from animals for an indefinite period, it is considered renewable. Similarly, gelatin and collagen are byproducts of the animal industry (skins, tendons, and bones) [99]. Although the process of raising animals is water-intensive and produces a large carbon footprint, using waste that would degrade in the environment can be both economical and sustainable.

Cellulose (nanocrystals and nanofibrils) can be obtained from wood resources [120] or cotton pulp [130,136]. Paper-based devices are being used for biodiagnostics and devices. Sustainable forestry and farming practices can make these resources viable. Increasingly, bacteria and algae are being explored for deriving these materials, including industrial production at low cost. All solid-state flexible supercapacitor was fabricated using bacterial nanocellulose obtained from *Gluconacetobacter xylinum* [122]. A paper-based battery was made using cellulose, which obtained from *Cladophora* green algae collected from the Baltic Sea [140]. Insect sources can provide materials such as shellac, which is a natural resin obtained from Lac beetles [264]. Marine resources are important in the extraction of chitin, chitosan, alginate, and agarose. A vast amount of crustacean waste is generated each year (shrimp shells, crab exoskeletons) and the development of value-added materials can provide an economic as well as an environmental benefit [157,160]. A glucose sensor was reported using β-chitin, where chitin was obtained from squid ink [165]. Squid and cephalopods also provide a source of melanin. DNA obtained from marine salmon can be used for various devices [234,238]. Note that often, the extraction of many of these materials (below) tend to require strong solvents, acids, bases, or energy intensive processing in comparison to plastics. Such industrial processing may therefore not be entirely environmentally benign. Another important consideration in the use of many nature-derived materials is the potential for batch-to-batch variation. As they are obtained from natural resources that may be intrinsically variable, the properties of the materials extracted may also be slightly altered. As large quantities of materials are adapted for industrial manufacture of devices, this will have to be addressed with tight quality control and characterization of feedstocks.

### 7.2 Extraction and solubility

A key step in the utilization of raw materials derived from natural sources involves processing them for use with device fabrication protocols. Unlike many synthetic materials, nature-derived materials are not easily processable in their normal forms (e.g. wool, hair, or feathers to yield keratin), and must undergo modifications before they can be used. This involves extraction and solubilization to form powders or solutions that may be used to fabricate device architectures. For instance, silkworm cocoons are boiled in sodium carbonate to remove the glue protein sericin, leaving behind SF fibers. The SF is solubilized in LiBr, and purified through centrifugation and dialysis. The resulting solutions can be lyophilized or cast to form thin-film substrates supporting electrode arrays or nanowires. Extraction of agarose from seaweed includes cleaning, chemical pretreatment, extraction, filtration, bleaching, and dewatering. It is then lyophilized into a white powder soluble in boiling water [184]. Keratin sources are washed with detergents and water, followed by extraction via alkaline hydrolysis. The alkaline agents break disulfide bonds resulting in water-solubility. Collagen is usually extracted from tissues using organic solvents [86]. DNA extraction from marine salmon involves homogenization, enzymatic treatment, carbon treatment for decolorization, filtration, and acetone precipitation [234]. These processes may themselves be energy intensive, which is an important consideration.

Once extracted, solubility of the materials is an important factor toward processing. Often the same material from different sources (e.g. keratin from feathers, skin) may exhibit different solubility properties. Polar aprotic solvents such as DMSO, DMF, FA, MFA are commonly used in the dissolution of agarose. Different solvent systems are used to dissolve cellulose (e.g. *N*-methylmorpholine-*N*-oxide, LiCl/*N*,*N*-DMAc, aqueous NaOH, alkali/urea, and tetra butyl ammonium fluoride/DMSO). 5–7% of LiCl in DMAc and NMP along with alkali/urea are some common solvents used for the dissolution of chitin. Melanin from squid ink may be dissolved in DMSO, NaOH, and NH_4_OH [252,253]. Indigo also suffers from extremely low solubility because of inter- and intramolecular hydrogen bonding and π–π stacking, and is soluble in small amounts in DMSO, DMF, and similar polar protic solvents [264].

Acids may be used for direct dissolution (e.g. chitosan, collagen in dilute acids, microcrystalline cellulose and nanocrystalline cellulose in HCl and sulfuric acid) [94]. Chitin is often acid hydrolyzed into low-molecular-weight chitin improving solubility. Photocrosslinkable chitin (PC) obtained by dissolving hydrolyzed chitin in 5% LiCl in DMAc, is soluble in formic acid and HFIP [285]. The solubility of α-chitin in HFIP allows it to be combined with other biomaterials such as SF for the realization of hybrid films with enhanced properties [159]. Often, materials such as cellulose are converted into processable derivatives such as sodium carboxymethyl cellulose and cellulose acetate [132]. Water soluble dopamine–melanin has been prepared via self-oxidation induced polymerization [256]. Ionic liquids have emerged as attractive solvents for a variety of materials, namely agarose, chitin, cellulose, and collagen [95].

Some natural materials such as silk sericin are water soluble in their native forms, making them processible using benign techniques such as low temperature and fully aqueous processes [67]. This is useful to form composites with biomolecules such as proteins, enzymes, and cells in bioactive devices. For instance, a composite with PEDOT:PSS and GOx was used to form a biosensor [68,69]. Water solubility implies faster degradation or dissolution in an aqueous/*in vivo* environment, and is therefore not always a benefit. For instance, the solubility of gelatin in water needs to be offset by use of crosslinkers, to be suitable for devices in physiological environments. DNA has been commonly modified with CTMA to form a water insoluble DNA-lipid complex that is soluble common organic solvents [234].

### 7.3 Processing and fabrication

Depending on the intended form factors and device architectures, various fabrication techniques have been covered above. It may be noted that many examples involve the biomaterials being used as substrates for different components or for printed circuitry. Most nature-derived materials may be destroyed by harsh solvents (e.g. developers, etchants), or high temperatures used in conventional clean room fabrication. Thus, integration with high resolution tools and high throughput manufacture are still under intensive research before such devices can reach the market.

Thin films and substrates are formed by techniques such as drop-casting, solvent-casting, spin coating, or blade coating. Film solubility can be altered with MeOH or EtOH, or water/thermal annealing [47,48]. SA solutions have been fabricated into thin films through drop-casting [183]. Drop-casting directly on top of functional components results in a thin film with embedded components (e.g. SF on top of Ag interdigitated electrodes [47], Mg resistor or graphite microscale patterns [48,50], aqueous solutions of keratin drop-casted directly over interdigitated electrodes [78] or to form dielectric layers in metal—insulator--metal and OTFT devices [75]). In another example, squid pen chitin in HFIP solution was the ink in an airbrush, which was used to spray chitin ink on PDMS molds, gratings, and pillars [176]. Although simple, drop-casting on top of irregular components may lead to uneven films without good adherence to underlying layers. Differences in evaporation rates can lead to uneven thickness, due to solution cohesion and pooling. Spin-coating results in uniform thickness, making it more advantageous. However, high spin speeds and fast drying times can affect the crystallization or assembly of the materials. There is also wasted material associated with spin-coating [57,58].

Solvent casting and spin coating remain common techniques for the fabrication of collagen films, although electrospinning, electrochemical polymerization, and aggregation have also been reported [98]. Fibrous architectures have been formed by various spinning methods (e.g. electrospun collagen [98,101], AgNW/fibroin composite fibers using wet-spinning [64], SA wet-spun in a CaCl_2_ solidification bath [182]) Metal components on films have been formed by sputter-coating (e.g. ultrathin Au or Ag on chitin [180]), e-beam deposition (e.g. metal on collagen [94,95]), contact-printing (e.g. Au/Cr electrode arrays, crystalline Si nanomembrane transistors, graphene on SF [[44], [45], [46],^49^]), or printing (e.g. carbon ink on agarose [191], metal on cellulose [[119], [120], [121]]). GaN has been introduced onto cellulose substrates using multistep processes involving e-beam deposition, sacrificial layer removal, reactive ion etching, and finally printing [286]. Indigo has been deposited on various substrates by evaporating purified indigo from a hot-wall epitaxy source at low pressure [264]. When combined with techniques such as e-beam, it is important to maintain low temperatures (~30 °C) to prevent biomaterial damage. For metals on films, deposition of thin layers can further improve the flexibility of the entire device.

The use of conducting polymers as discussed in several aforementioned examples has been particularly attractive because they can be combined with natural materials under relatively benign conditions (e.g. cellulose and collagen combined with PPy and PANI via *in situ* polymerization [99]) or by simple solvent processing (e.g. PEDOT:PSS composites with silk sericin). The choice of a proper oxidant and dopant can play a role on the electrochemical properties of the composite. The use of photolithography to form high resolution architectures has become possible via the use of photocrosslinkable variants including gelatin methacrylate (GelMA), silk photofibroin, photochitin, and keratin using solvents such as formic acid and HFIP. After exposure to UV light through a patterned photomask, various micropatterned architectures and devices have been reported [68,82].

### 7.4 Degradation

**Fig. 10:**
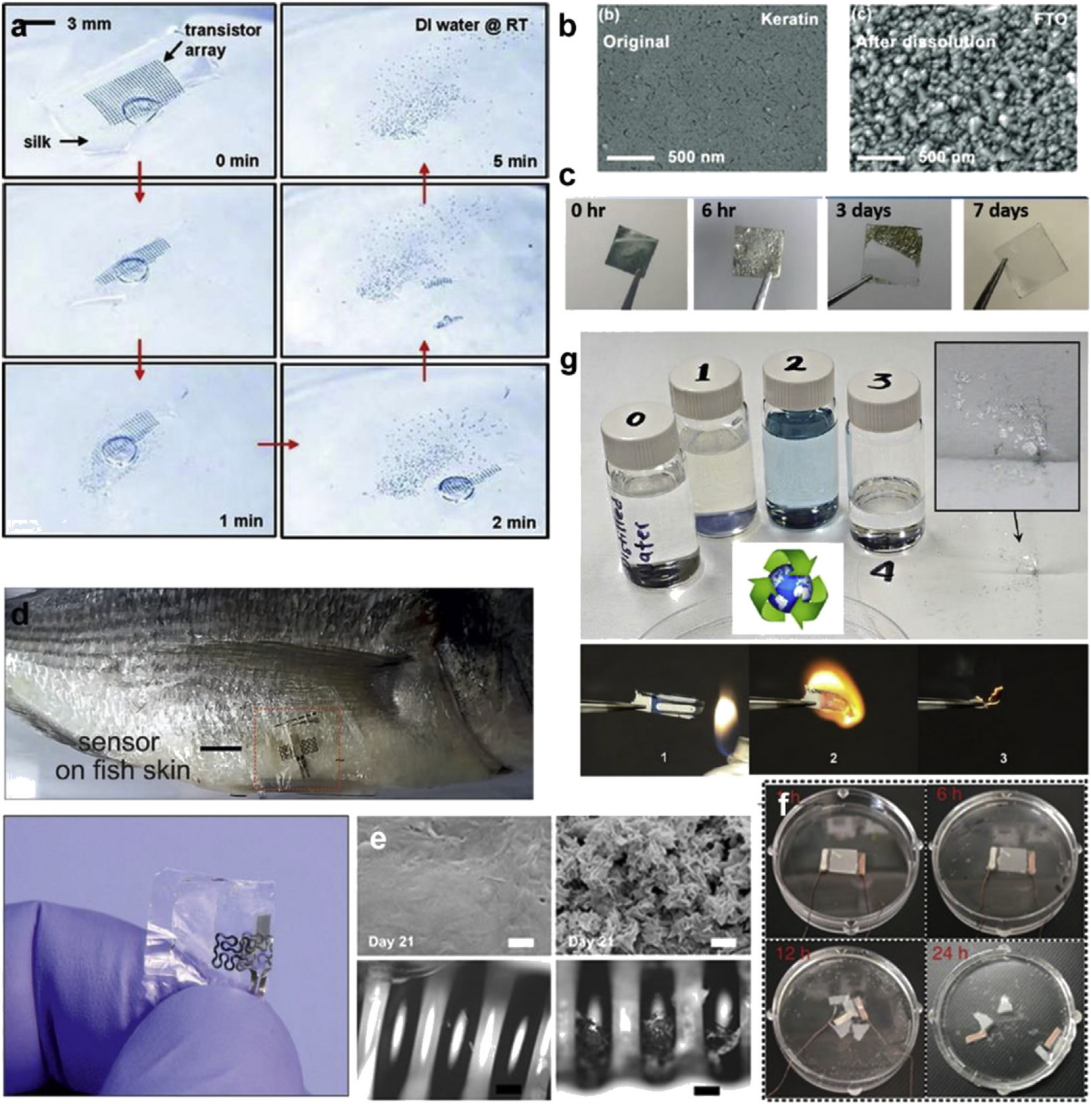
**Degradation modes**. **(a)** Optical images of disintegration and dissolution of an array of fully formed transient transistors on film of silk. The n-channel MOSFETs have total thicknesses of <2 μm, including a base layer of thermal SiO_2_ (≈1 μm), Mg electrodes, SiO_2_ gate dielectric, and Si as the semiconductor. Dissolution occurs in deionized water at room temperature [292]. **(b)** Keratin thin film coated on FTO/glass substrate observed using SEM after dissolution in water [77]. **(c)** Dissolution test of PCDTPT/keratin TFT on keratin substrate in ammonium hydroxide solution [80]. **(d)** Sensors fully composed of biodegradable materials including Mg, silicon dioxide and nitride, and a compostable flexible polymer (i.e., Ecoflex™) placed on fish skin to track temperature during storage [216]. **(e)** PEDOT:PSS--sericin ink-printed electrodes on a silk fibroin substrate degraded in a protease solution [197]. **(f)** Degradation of the sensor based on Ag NFs/SF films in 5% papain solution [63]. **(g)** Vials (#0–3) and filter paper (#4) illustrating the separation of solar cells into their major components by immersion in water and chlorobenzene. The inset is a close-up of the solid waste left on the filter paper showing residues of Ag and MoO_3_. Time lapse sequence of three frames illustrating the ignition of solar cells on CNC substrates: #1: an image of a solar cell before burning; #2: while burning; #3: after burning. Burning lasted less than 2 s [123]. (*All images used with permission*).

Degradation may be considered in two contexts, namely biodegradation *in vivo* by enzymatic or hydrolytic action or, degradation in the environment after use. Some typical degradation methods of biodevices are shown in Fig. 10. The controllable degradation of natural biomaterials *in vivo*, presents opportunities for transient and implantable devices. Dissolution may occur in aqueous media, specialized solvents, or via enzymatic degradation. For instance, silk films have been shown to dissolve within hours in saline [44,63,66]. Silk films are also proteolytically degradable in the span of days to weeks [[68], [69], [70], [71]]. Some devices exhibited stability in serum for up to a week, which could be useful for long-term sensing applications [55,71]. Solubility of different components of devices on different timescales may be a challenge. For example, in a layered device submerged in PBS at 37˚C, the Mg top electrode dissolved in 3 min, the silk film in 3 h, and the W bottom electrode in 24 h [57]. While an outer envelope of a less-soluble compound can extend device life, it could also hinder function. Degradability can be altered via surface treatment, solvent treatment, or blending with other materials [47,52,60,61].

Materials such as keratin, cellulose, and chitin are degradable to varying extents. Keratin is considered to be insoluble in water, which could be useful for long-term applications where transience is not desired. Patterned keratin films were stable in PBS over 7 days, and degradable in 15 days with the addition of proteases [82]. Modification of the solvent or the material itself can be used to tune solubility as well (e.g. 30 min to several weeks) [77,79]. An alkaline solution (28–30% ammonium hydroxide solution) at room temperature was also used to dissolve keratin substrate and dielectric layers in a device over the course of 1 week [80]. In an interesting consideration, fish have been shown to consumer keratin-coated Si wafer [75].

A piezoelectric chitin film used as a speaker was shown to degrade in the presence of chitinase enzyme within 8 days [159]. A chitin-derived electronic circuit was almost completely degraded in soil within 30 days [160]. A chitin--SF hybrid film that has been used as a substrate for glucose-sensing lens, film-type wireless heater, and a transparent display cover plastic was made to degrade in the presence of hydrolytic enzymes for 10 days with a degradation of 40–60% [162]. Composting or burning of devices made of cellulosic materials is another interesting option to dispose of the devices [123]. Mitigating the problem of ‘e-waste’ and improving environmental sustainability is a driving force for use of natural materials. As materials that can eventually degrade, they present alternatives both in terms of green processing but also preventing landfill pollution. Overall, various routes exist for device degradation by avoiding materials that would persist in the environment for thousands of years.

### 7.5 Biocompatibility

Biocompatibility of materials is another critical aspect of device safety. Before their usage in or on humans, each device needs to be tested to ensure that the constituent materials do not cause adverse reactions. We note that simply because a material is nature-derived, there is no guarantee of their biocompatibility. Each material needs to be carefully studied for their specific application using standardized tests (e.g. ISO: 10,993) that outline laboratory and animal tests and end-points. While it is preferred that these tests be performed using the final, sterilized device materials, companies may justify testing performed on the raw device material is representative of the final, sterilized form. Both the bulk device material, as well as the specific processing and finishing steps used on the material in a particular device need to be investigated. It is also important to assess any by-products created by the device either chemically or mechanically.

In the previous paragraph, we have outlined several examples of how many of the nature-derived materials tend to have excellent biocompatibility characteristics. For instance, silk proteins are notable for their biocompatibility through *in vitro* and *in vivo* studies [49,60]. *In vivo* studies of silk film--based devices in feline and rat brains did not lead to inflammation or obvious cytotoxicity [44,66]. Silk sensors have also been fixed to human skin without irritation [47,50,54,61,63,65,68,287]. The biocompatibility of keratin devices and composites *in vitro* and *in vivo* still remains to be fully elucidated. Studies with model bone marrow--derived mesenchymal stem cells showed that there was no cytotoxicity associated with keratin films, and that cells exhibited preferential adhesion on these substrates due to keratin's natural RGD adhesive ligands [81,82].

Biocompatibility studies were performed on PEDOT:gelatin films, which were shown to promote adhesion and growth of bovine brain capillary endothelial cells [288]. PC12 cells showed viability on PPy-chondroitin sulfate (CS) films modified with collagen type I [289]. *In vivo* tests were performed for 4 weeks by implanting collagen-modified glucose sensors in rats [290]. Chitin-silk and chitin-GOx membranes were studied in live rabbits and using L-929 mouse fibroblasts showing *in vivo* and *in vitro* sensitivity and biocompatibility tests [162]. The biocompatibility of melanin thin films was tested *in vivo* using Schwann cells. *In vivo* tests were performed by placing the films in rats for 8 weeks [252]. Note that in many cases, the biomaterials are combined with other biomaterials (forming fully organic devices) or dopants (e.g. conducting polymers, CNTs), or various metals (e.g. Au, Pt) that may be deposited on the support substrates. In the case of composites, the properties of all components need to be investigated, both in terms of degradation and biocompatibility. Thus, several natural biomaterials have been shown to be very promising in terms of their biocompatibility and their potential for human use, either *in vivo* as implanted devices or as external, wearable applications.

## 8 Conclusions

Nature provides an abundance of ideas both in terms of inspiration (biomimetics) and as a source of materials for green electronics and functional device fabrication. There are a wealth of nature-inspired and nature-derived substrates, dielectrics, and semiconductors that have proven to be useful as support structures and active forms for electronic and optoelectronic devices. Green electronics provides an efficient and ‘natural’ solution for e-waste management and environment protection. Furthermore, green electronics present immense potential for biomedical applications in terms of functionally transient and implantable devices. Although these materials display a palette of unique characteristics, it is clear that performance parameters, including electric conductivity, mechanical flexibility, as well as controllable biodegradability and biocompatibility, still need be further improved to make them viable competitors to conventional electronic devices. Improving green processing of natural feedstocks and the development of suitable protocols are needed to ensure batch-to-batch consistency so that production of devices can be synchronized globally. Opportunities abound for research—from the identification and characterization of new materials, novel device designs, optimization of electrochemical or optoelectronic properties, to the fabrication of functional prototypes for translation to the marketplace. Drawing from this unique interdisciplinary research can help facilitate realization of this vision.

## Declaration of competing interest

The authors declare that they have no known competing financial interests or personal relationships that could have appeared to influence the work reported in this paper.
